# Cortical Gradients Support Mental Time Travel into the Past and Future: Evidence from Activation Likelihood Estimation Meta-analysis

**DOI:** 10.1007/s11065-025-09662-w

**Published:** 2025-05-01

**Authors:** Alice Teghil, Martin Wiener, Maddalena Boccia

**Affiliations:** 1 Department of Psychology, “Sapienza” University of Rome, Via Dei Marsi, 78, 00185 Rome, Italy; 2 Cognitive and Motor Rehabilitation and Neuroimaging Unit, IRCCS Fondazione Santa Lucia, Rome, Italy; 3 Department of Psychology, George Mason University, Fairfax, VA USA

**Keywords:** Episodic memory, FMRI, Prospection, Episodic simulation, Functional gradients, Meta-analysis

## Abstract

A longstanding issue concerns the extent to which episodic autobiographical memory (EAM) and episodic future thinking (EFT) are the expression of the same cognitive ability and may be dissociated at the neural level. Here, we provided an updated picture of overlaps and dissociations between brain networks supporting EAM and EFT, using Activation Likelihood Estimation. Moreover, we tested the hypothesis that spatial gradients characterize the transition between activations associated with the two domains, in line with accounts positing a transition in the relative predominance of their features and process components. We showed the involvement of a core network across EAM and EFT, including midline structures, the bilateral hippocampus/parahippocampus, angular gyrus and anterior middle temporal gyrus (aMTG) and the left superior frontal gyrus (SFG). Contrast analyses highlighted a cluster in the right aMTG significantly more activated during EFT compared with EAM. Finally, gradiental transitions were found in the ventromedial prefrontal cortex, left SFG, and bilateral aMTG. Results show that differences between EAM and EFT may arise at least partially through the organization of specific regions of common activation along functional gradients, and help to advocate between different theoretical accounts.

**Supplementary Information:**

The online version contains supplementary material available at 10.1007/s11065-025-09662-w.

## Introduction

The possibility to “mentally travel” into the past, as well as into the future, represents one of the most extraordinary features of human experience. Endel Tulving (2002) defined *chronestesia* as a specific form of consciousness, entailing the awareness of the temporal dimension of one’s own life. Tulving further suggested that this kind of experience is essential to mental activities such as the recollection of personal past events, and the anticipation and imagination of future happenings (Tulving, 2002). Whereas the recollection of events from our own past falls within the domain of *episodic autobiographical memory*, the simulation, anticipation, and imagination of events collocated in a personal future is broadly referred to as *episodic future thinking* (Schacter et al., 2017).

A longstanding issue in cognitive neuroscience concerns the degree to which episodic autobiographical memory (EAM) and episodic future thinking (EFT) are basically the expression of the same, core cognitive ability (i.e. mental time travel), or rather rely more strongly on different process components, and to which extent their neural correlates may be dissociated (Clayton et al., 2003).

Starting from single case reports of a concurrent impairment of amnesic patients in remembering past events and imagining personal future episodes (Tulving, 2002; Klein et al., 2002), neuropsychological studies provided evidence that bilateral hippocampal damage affects not only episodic memory, but also the ability to imagine new events (Hassabis et al., 2007). Patients with amnesia due to medial temporal lobe lesions are similarly impaired in producing specific details when remembering past events and when imagining future ones, despite a normal performance in producing narratives when details are externally available (Race et al., 2011). Focal lesions to the ventromedial prefrontal cortex (vmPFC) also result in a reduced production of episodic details for both the recall of autobiographical events and the imagination of self-related future ones (Bertossi et al., 2016a). A similar concurrent reduction in the specificity of EAM and EFT has been reported following traumatic brain injury (Rasmussen & Berntsen, 2014) and in patients with amnestic Mild Cognitive Impairment and Alzheimer’s Disease (Gamboz et al., 2010; Addis et al., 2009a), as well as in Korsakoff’s syndrome (Janssen et al., 2022). This body of evidence parallels findings in healthy individuals, showing that EAM and EFT develop at the same time during childhood (Busby & Suddendorf, 2005) and are similarly affected by aging (Addis et al., 2008). Moreover, valence and temporal distance of the events (D’Argembeau and Van der Linden, 2004) as well as individual variations in visual mental imagery (D’Argembeau and Van der Linden, 2006) similarly influence mental time travel into the past and future, further suggesting their reliance on shared mechanisms.

Neuroimaging studies also show substantial overlap between brain regions supporting EAM and EFT, with both activating a network involving the posterior cingulate cortex, the angular gyrus, the medial temporal lobes and anterior middle temporal gyrus, as well as the superior and medial frontal cortex (Teghil et al., 2021; Stawarczyk and D’Argembeau, 2015; Cona et al., 2023a). Using activation likelihood estimation (ALE), Benoit and Schacter (2015) showed that memory for autobiographical events and episodic simulation (including the imagination of fictional, possible future, and counterfactual or alternative events) involve a core network including the hippocampus, the parahippocampal cortex, the anterior cingulate cortex, the rostral and ventral medial prefrontal cortex, the dorsomedial prefrontal cortex, the posterior cingulate and retrosplenial cortex, the lateral temporal cortex, as well as the posterior and inferior parietal cortex, the superior temporal lobe, the right dorsolateral prefrontal cortex and the left inferior frontal gyrus (Benoit & Schacter, 2015).

Based on the lines of evidence mentioned above, different accounts have been proposed suggesting a substantial overlap between cognitive and neural processes supporting EAM and EFT (Addis, 2020; Hassabis & Maguire, 2007; Schacter & Addis, 2007; Suddendorf & Corballis, 1997). Buckner and Carroll (2007) proposed that the core neural network involved in both EAM and EFT supports self-projection, suggesting that similarities between EAM and EFT may be explained by the fact that both rely on the ability to mentally project the self into a simulation of another time, place or perspective (Buckner & Carroll, 2007). Similarly, Hassabis and Maguire (2007) proposed that similarities between EAM and EFT may be accounted for positing that they both depend on scene construction, that is the generation and maintenance of a coherent spatial scene (Hassabis & Maguire, 2007). Suggesting an even more substantial overlap between EFT and EAM, the constructive episodic simulation hypothesis proposes that the simulation of our personal future is supported by episodic memory, as the representations of events that may happen in the future are generated through the extraction and recombination of elements from previous experiences (Schacter & Addis, 2007). Also, similar cognitive processes would be involved during past and future event construction, including mental imagery and self-referential processing (Addis et al., 2009b). More recent elaborations of this account suggest that constructive episodic simulation may be conceived as a neurocognitive system, supporting both memory and imagination (Addis, 2020). Within this framework, a single neural system would support the domain-general capacity to use perceptual and conceptual information from previous experience to generate simulations, that would be mapped in multimodal cognitive spaces through the essential contribution of the hippocampus (Addis, 2020).

Nonetheless, differences have also been reported between EAM and EFT at the phenomenological and neurocognitive level. Simulated future events are characterized by less contextual details (D’Argembeau & Van der Linden, 2004; 2006; Bertossi et al., 2016a) and require more effort to be produced (Gilmore et al., 2018; McDonough & Gallo, 2010) compared with past memories. Also, a preserved ability to imagine future events despite impaired episodic autobiographical memory specifically for recent events has been reported in patients with hippocampal and medial temporal lobes lesions, suggesting that EFT may depend more strongly than EAM on regions other than the hippocampus (Squire et al., 2010). On the other hand, an impairment of EFT with relatively preserved EAM has been reported in patients with semantic dementia, showing that the ability to retrieve past episodes is not enough to support the imagination of novel future events (Irish et al., 2012a, 2012b). Based on these latter findings, it has been proposed that conceptual information is particularly relevant for the imagination of novel future events, since it provides an amodal framework into which episodic details may be integrated (“semantic scaffolding hypothesis”) (Irish & Piguet, 2013; Irish et al., 2012a, 2012b). According to this view, semantic memory is as important as episodic memory in supporting EFT (Irish et al., 2012a).

Neuroimaging studies further provided evidence that a set of brain regions including the dorsolateral and dorsomedial prefrontal cortex, the posterior cingulate cortex and precuneus, the right hippocampus and the bilateral lateral temporal cortex and posterior inferior parietal lobule are more activated during EFT compared to EAM (Benoit & Schacter, 2015). This suggests that regions of the core network may be differently recruited depending on whether the constructed event is past or future (Schacter et al., 2008). Differences observed between activation patterns associated with future and past events have been mainly explained in the light of the constructive episodic simulation framework, suggesting that imagining the future requires the combination of details extracted from different episodes into a novel event, thus imposing higher demands on the hippocampus and on frontoparietal control networks (Benoit & Schacter, 2015). It has been further proposed that other phenomenological differences between EAM and EFT may be explained by a different degree of associative strength and by the differential involvement of schemas, since remembering the past would entail maximum associative strength and minimum schema reliance, whereas the opposite would be true for future imagination (Addis, 2020).

Overall, although many lines of evidence suggest a tight link between EAM and EFT, it is still not clear how differences between them may be accounted for by their organization at the neural level. Thus, in the present study, we aimed to provide an updated picture of neuroanatomical differences and convergences between EAM and EFT. To this aim, we adopted a meta-analytic approach, using Activation Likelihood Estimation (ALE) to quantitatively assess the overlap between brain networks supporting EAM and EFT, as well as differences between such networks. Importantly, in the only previous quantitative meta-analysis assessing the overlap between brain regions supporting EAM and EFT, the authors included studies requiring not only the generation of possible future events, but also of counterfactual and fictitious episodes (Benoit & Schacter, 2015). Thus, in contrast with the defining features of EFT (Addis et al., 2007), included experiments also tested atemporal events and events with different degree of plausibility. Moreover, only experiments reporting joint activation between EAM and EFT were considered, therefore limiting the number of included contrasts. The first aim of this study was thus to specify common and specific brain activations associated with remembering episodes from one’s own past (EAM) and with the imagination of possible events in one’s own future (EFT).

Regions of the core network supporting EAM and EFT strongly overlap with the Default Mode Network (DMN) (Benoit & Schacter, 2015), that has been implicated in various forms of complex cognition requiring the disengagement from the external environment, including semantic and episodic memory, self-referential processing and social cognition (see Andrews-Hanna et al., 2014; Smallwood et al., 2021, for reviews). The DMN has been proposed to represent the culmination of a representational gradient, progressing from sensorimotor towards transmodal association regions (Margulies et al., 2016; Smallwood et al., 2021). Gradient organization—defined as axes along which cortical features vary in a spatially organized manner (Huntenburg et al., 2018)—has been suggested to work as a general organizational principle along the cortex, supporting the processing of information at increasing level of abstraction (Huntenburg et al., 2018; Margulies et al., 2016). Cortical gradients have been demonstrated in different domains, including the interaction between time, space and number processing (Cona et al., 2021, 2023b), in line with the possibility that they may support spatially organized functional specialization along the cortex. Thus, consistent with the proposal that differences between EAM and EFT may be understood from the perspective on a transition in the relative predominance of their features and process components (Addis, 2020), as a second aim we tested the hypothesis that spatial gradients characterize the transition between brain activations associated with EFT and EAM.

To these goals, we first performed two individual ALE meta-analyses on brain correlates of EAM and EFT. Then, we performed conjunction and contrast analyses to identify regions jointly and differentially involved in the two processes. Finally, we tested the presence of functional gradients in areas of convergence between EAM and EFT.

Besides allowing to characterize differences between brain networks supporting EAM and EFT in a more fine-grained manner, such an approach can also help to advocate between different theoretical accounts, specifying brain regions which functional properties appear to vary in parallel with the transition from past-to-future event processing.

## Meta-analysis

### Inclusion Criteria for Papers

The search for relevant literature was performed in a systematic manner, using the PubMed and Scopus databases. The following a priori inclusion criteria were specified: (1) only articles reporting studies performed using functional magnetic resonance imaging (fMRI) were included; (2) only articles reporting whole-brain analyses were included; thus, articles reporting only region of interest (ROI) analyses were excluded; (3) articles had to clearly report coordinates of activation foci in either Montreal Neurological Institute (MNI) or Talairach reference space; (4) only group studies were included; (5) we only included studies not involving any manipulation of the psychophysical conditions of volunteers (including pharmacological manipulations, psychotherapeutic interventions or other manipulations); (6) we did not include papers and/or single experiments involving aged volunteers (> 65 years), as confirmed by the age range and/or mean and standard deviation of participants’ age; (7) only individual experiments including a visuo-perceptual/motor control condition were included; (8) only individual experiments reporting the results of univariate analyses were included; (9) studies had to involve either the recollection (episodic autobiographical memory) or the imagination (episodic future thinking) of personal life events. A PRISMA (Preferred Reporting Items for Systematic Reviews and Meta-Analyses) (Page et al., 2021) diagram illustrating selection procedures and included studies can be found in Supplementary Materials (Fig. S1, Fig. S2, Table S1 and Table S2).

For the ALE meta-analysis on EAM, we included all individual experiments included in a previous meta-analysis (Teghil et al., 2021) that satisfied the a priori criteria specified in the current study. This led to the exclusion of 15 experiments from 4 papers that reported the results of multivariate analyses (Rabin & Rosenbaum, 2012; Addis et al., 2004, 2012; McCormick et al., 2015). A further systematic literature search was performed in September 2022, using the strings: “autobiographical memory AND fMRI” and “autobiographic memory AND fMRI”. This search produced 1051 articles. After duplicates were removed, 848 articles were screened from title and abstract. The final selection according to our a priori inclusion criteria led to the selection of 11 papers and 18 experiments. Thus, the meta-analysis on EAM was performed on a total number of 82 experiments from 44 papers (Table S1).

For EFT, a systematic search was performed in September 2022 using the strings: “episodic future thinking AND fMRI”. The search produced 146 journal articles. After duplicates were removed, 73 articles were screened from title and abstract. The meta-analysis on EFT was performed on a total number of 32 experiments from 17 papers. The complete list of included contrasts is reported in Supplementary Materials (Table S2). A PRISMA 2020 checklist is included in Supplementary Materials (Table S3). A summary of risk of bias assessment of included studies is provided in Supplementary Materials (File S4).

### Activation Likelihood Estimation

Activation Likelihood Estimation (ALE) meta-analysis evaluates the spatial clustering of activation foci from different experiments that involve the same cognitive function or domain. In brief, ALE models activation foci as probability distributions centered at the specific coordinates reported, and tests whether the clustering of such activation foci is higher than what expected under the random distribution of the same activation foci. It results in a thresholded ALE map representing the combined activation probability for each voxel to contain at least one of the activation foci.

We first performed two general meta-analyses on experiments selected for the EAM and EFT domains. We then performed a conjunction analysis between EAM and EFT, and two contrast analyses (EAM > EFT and EFT > EAM) to assess common and specific activations associated with the two domains. ALE analyses were performed using GingerALE 3.0.2 (brainmap.org), with MNI coordinates. Talairach coordinates were automatically converted into MNI space using GingerALE. Within-experiment effects were minimized by taking the maximum probability associated with any focus reported in the experiment, according to the non-additive procedure introduced by Turkeltaub and colleagues (2012). The ALE values for each voxel were calculated according to the standard procedure (Eickhoff et al., 2009). The Full-Width Half-Maximum (FWHM) value was calculated automatically, being empirically determined (Eickhoff et al., 2009). All the thresholded ALE maps were computed using *p* values from the previous step, a cluster-level inference at the 0.05 significance level with 1000 threshold permutations and a cluster-forming threshold of *p* < 0.001 uncorrected (Eickhoff et al., 2016a, 2016b).

In order to assess the possible impact of publication bias on the results of the two meta-analyses, we further implemented the procedure based on “Fail-Safe N” (FSN) calculation, proposed by Acar and colleagues (2018). This procedure allows to assess the robustness of ALE results against the file drawer effect, that is the tendency for studies with significant results to be more likely published than those with non-significant results. For ALE, this can be tested by adding to the meta-analysis a specified number of null studies, that is, studies report foci randomly distributed throughout the brain. The range of neuroimaging studies remaining unpublished due to non-significant results has been estimated to range between 5 and 30% of published studies (Samartsidis et al., 2020). Here, we quantified the robustness of our results applying the upper bound of this estimate, introducing the 30% of noise studies for each meta-analysis (EAM and EFT).

### Gradient Analysis

To investigate potential gradients of activation likelihood, we conducted a gradient ALE analysis (Cona, et al., 2021, 2023b). First, difference maps were calculated by subtracting ALE values from the EAM analysis from the EFT analysis, where positive values represent greater likelihood for EFT and negative values represent greater likelihood for EAM; values around zero would thus represent equal probability for either meta-analysis whereas gradients would be located in continuous transitions from positive to negative numbers.

To determine the stability and reliability of gradients, we used a permutation approach adopted previously (Cona, et al., 2021, 2023b). To determine reliability, a null distribution of ALE difference scores was generated by taking the initial coordinates for EAM and EFT meta-analyses and randomly scattering them across the brain, then generating unthresholded ALE maps for each, then again subtracting EAM from EFT; this process was repeated 1000 times to generate a null distribution representing the random occurrence of gradients. A similar approach was used to estimate stability, wherein we selected a random 70% of the coordinates for EAM and EFT meta-analyses, generated a new ALE map for each and again subtracted EAM from EFT, with another 1000 repetitions of the process performed. Reliability could thus be assessed by comparing the gradients observed in the “full” meta-analysis to difference maps at those same locations in the null distribution, whereas stability could be measured by examining gradients in the 70% dataset; if the gradients observed were robust, they would be larger than that observed in the null dataset, and should be consistent in the 70% dataset, indicating they were not due to either randomness or small number of studies (Vos de Wael et al., 2020). The gradient analysis was performed using custom MATLAB scripts (https://www.mathworks.com).

## Results

### General Meta-analysis on EAM

**Fig. 1:**
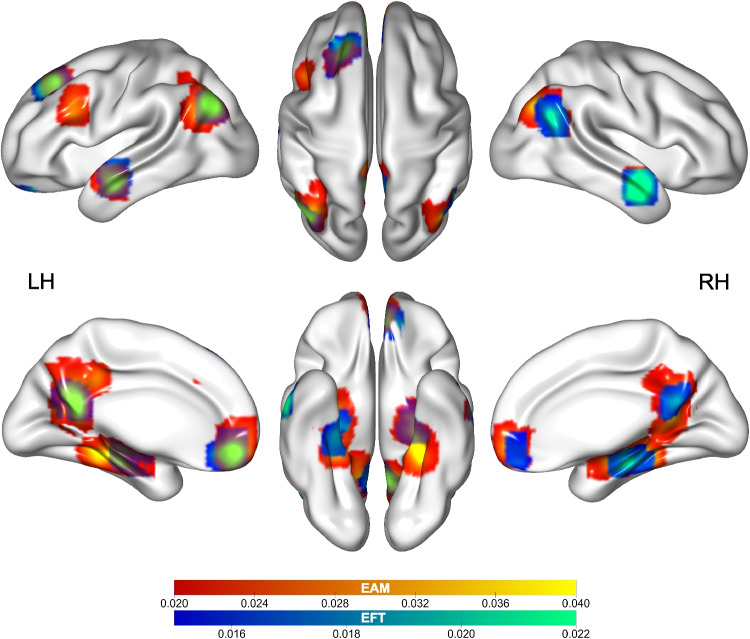
Results of the individual ALE meta-analyses on episodic autobiographical memory (EAM, shown in red-to-yellow) and episodic future thinking (EFT, shown in blue-to-green). LH, left hemisphere; RH, right hemisphere

Results of the general meta-analysis on EAM are reported in Table 1. In line with previous meta-analyses (Boccia et al., 2019; Teghil et al., 2021), a first cluster was found in the posterior cingulate cortex (PCC) extending to the precuneus (pCu). Activations were also observed in the bilateral parahippocampal gyrus (PHG), extending to the hippocampus (HC) in the right hemisphere, and in the bilateral anterior middle temporal gyrus (aMTG) and angular gyrus (AG). The inferior (IFG) and superior (SFG) frontal gyri were also activated in the left hemisphere. Further clusters of activation were observed in the ventromedial prefrontal cortex (vmPFC), extending to the anterior cingulate cortex, as well as in the right posterior cerebellum (Fig. 1).

**Table 1:** Results of the ALE meta-analysis on EAM. For each cluster, we report region label, hemisphere, cluster size (mm^3^), ALE value and MNI coordinates

Cluster	Region	Hemisphere	Volume (mm^3^)	ALE value	*x*	*y*	*z*
1	PCC/RSC	LH	16,320	0.07060164	− 4	− 58	24
		LH		0.06544434	− 8	− 56	16
		LH		0.04368008	− 4	− 42	36
		RH		0.04021281	14	− 52	12
		RH		0.02840995	10	− 48	22
		LH		0.02572772	− 4	− 68	38
2	PHG	LH	8480	0.05681535	− 24	− 26	− 14
		LH		0.05211847	− 28	− 38	− 12
		LH		0.02326592	− 28	− 30	− 26
3	AG	LH	7104	0.06990492	− 44	− 70	34
4	vmPFC/ACC	LH	6208	0.0568039	− 4	50	− 8
5	FG/PHG	RH	6000	0.06570642	26	− 36	− 14
		RH		0.04615029	24	− 22	− 16
		RH		0.04304995	26	− 14	− 18
6	AG	RH	5176	0.04244169	48	− 68	28
		RH		0.02746987	54	− 56	22
7	SFG	LH	1776	0.03669509	− 20	30	48
8	pCer	RH	1632	0.03853083	26	− 80	− 34
		RH		0.02486604	16	− 78	− 30
9	aMTG	LH	1520	0.04028198	− 58	− 4	− 16
10	IFG	LH	1272	0.03648405	− 44	18	28
11	aMTG	RH	1128	0.04112285	60	− 6	− 18

*PCC*, posterior cingulate cortex; *RSC*, retrosplenial cortex; *PHG*, parahippocampal gyrus; *AG*, angular gyrus; *vmPFC*, ventromedial prefrontal cortex; *ACC*, anterior cingulate cortex; *FG*, fusiform gurys; *SFG*, superior frontal gyrus; *pCer*, posterior cerebellum; *aMTG*, anterior middle temporal gyrus; *IFG*, inferior frontal gyrus; *LH*, left hemisphere; *RH*, right hemisphere

The FSN analysis showed that most of the highlighted clusters were robust against publication bias, remaining significant after adding the 30% noise studies to the meta-analysis. The only exception was for the left IFG cluster (cluster 10), that was no longer significant.

### General Meta-analysis on EFT

In the general meta-analysis on EFT (Table 2), activations were found in the PCC, as well as in the bilateral AG. The PHG and the HC showed clusters of activation respectively in the right and left hemisphere, with the HC cluster also extending to the left PHG. The aMTG was also bilaterally activated. Finally, clusters of activations were found in the frontal lobe of the left hemisphere, corresponding to the SFG and to the vmPFC, and in the anterior cerebellum (Fig. 1).

**Table 2:** Results of the ALE meta-analysis on EFT. For each cluster, we report region label, hemisphere, cluster size (mm^3^), ALE value and MNI coordinates

Cluster	Region	Hemisphere	Volume (mm^3^)	ALE value	*x*	*y*	*z*
1	vmPFC	LH	2632	0.03542178	− 6	46	− 14
2	PCC/RSC	LH	2272	0.03705081	− 2	− 54	18
		LH		0.01623805	− 12	− 64	22
3	aMTG	RH	2000	0.05038734	58	− 4	− 20
4	SFG	LH	1712	0.04021892	− 20	34	44
5	aMTG	LH	1664	0.04404118	− 60	− 6	− 16
6	AG	LH	1552	0.04518519	− 46	− 72	32
7	AG	RH	1176	0.02528057	52	− 62	24
8	PHG	RH	1152	0.02752917	26	− 20	− 18
9	HC/PHG	LH	1000	0.02237126	− 24	− 20	− 18
		LH		0.02096563	− 30	− 24	− 12
		LH		0.01730908	− 20	− 24	− 10
10	aCer	RH	856	0.02230889	4	− 56	− 46
		LH		0.02206821	− 4	− 52	− 42

*vmPFC*, ventomedial prefrontal cortex; *PCC*, posterior cingulate cortex; *RSC*, retrosplenial cortex; *aMTG*, anterior middle temporal gyrus; *SFG*, superior frontal gyrus; *AG*, angular gyrus; *PHG*, parahippocampal gyrus; *HC*, hippocampus; *aCer*, anterior cerebellum; *LH*, left hemisphere; *RH*, right hemisphere

After applying the FSN analysis, the left HC/PHG and the aCer clusters (clusters 9 and 10) were no longer significant, suggesting that results supporting their consistent involvement in EFT are less robust and might be affected by publication bias.

### Conjunction Analysis

**Fig. 2:**
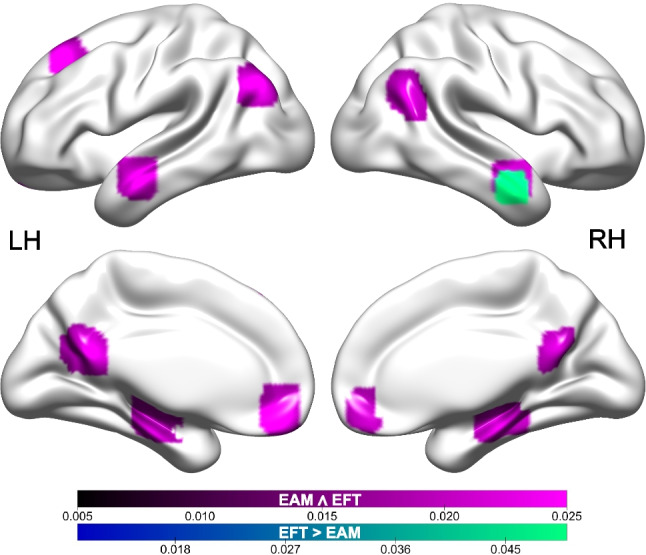
Results of the conjunction analysis between episodic autobiographical memory (EAM) and episodic future thinking (EFT) (shown in black-to-magenta) and of the contrast analysis EFT > EAM (shown in blue-to-green). LH, left hemisphere; RH, right hemisphere

Results of the conjunction analysis between EAM and EFT (Table 3) showed a shared network involving the left PCC and vmPFC on the medial brain surface, as well as the bilateral AG and aMTG and the left SFG on the lateral surface. Further clusters were found in the right PHG and in HC extending to the PHG in the left hemisphere (Fig. 2).

**Table 3:** Results of the conjunction and contrast analyses on EAM and EFT. For each cluster, we report region label, hemisphere, cluster size (mm^3^) and MNI coordinates

Cluster	Region	Hemisphere	Volume (mm^3^)	*x*	*y*	*z*
*EAM ∧ EFT*						
1	PCC/RSC	LH	2184	− 2	− 54	18
		LH		− 12	− 64	22
2	vmPFC	LH	1800	− 6	46	− 12
3	AG	LH	1392	− 46	− 72	32
4	aMTG	LH	1032	− 60	− 6	− 16
5	PHG	RH	952	26	− 20	− 18
6	aMTG	RH	896	58	− 6	− 18
7	HC/PHG	LH	864	− 24	− 20	− 18
		LH		− 30	− 24	− 12
		LH		− 20	− 24	− 10
8	SFG	LH	840	− 20	32	48
9	AG	RH	744	50	− 62	26
*EAM* > *EFT*						
No suprathreshold cluster						
*EFT* > *EAM*						
1	aMTG	RH	208	58	− 6	− 26

*PCC*, posterior cingulate cortex; *RSC*, retrosplenial cortex; *vmPFC*, ventromedial prefrontal cortex; *AG*, angular gyrus; *aMTG*, anterior middle temporal gyrus; *PHG*, parahippocampal gyrus; *HC*, hippocampus; *SFG*, superior frontal gyrus; *LH*, left hemisphere; *RH*, right hemisphere

### Contrast Analyses (EAM > EFT, EFT > EAM)

The contrast EAM > EFT did not show any suprathreshold cluster of activation. Concerning the opposite contrast, a single cluster in the right aMTG (Table 3) showed stronger activation during EFT compared to EAM (Fig. 2).

### Gradient Analysis

**Fig. 3:**
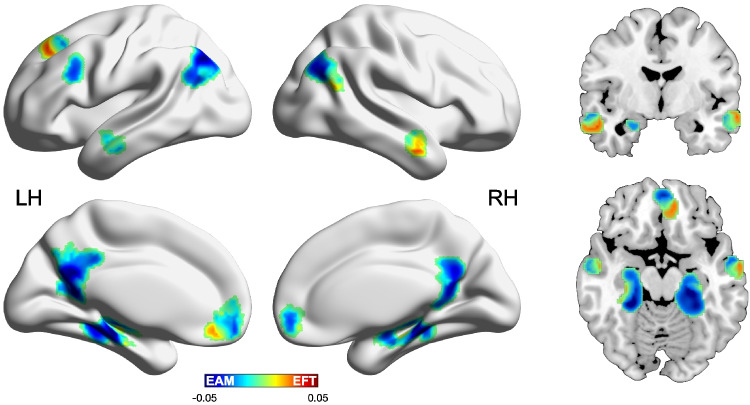
Gradient analysis. Left: surface visualization of the overlap between autobiographical memory (EAM) and episodic future thinking (EFT) meta-analyses. ALE values for EAM were set to negative numbers and added to ALE values from the EFT meta-analysis. Right: a coronal and axial slice displaying the same values, highlighting the left and right middle temporal gyrus gradients

**Fig. 4:**
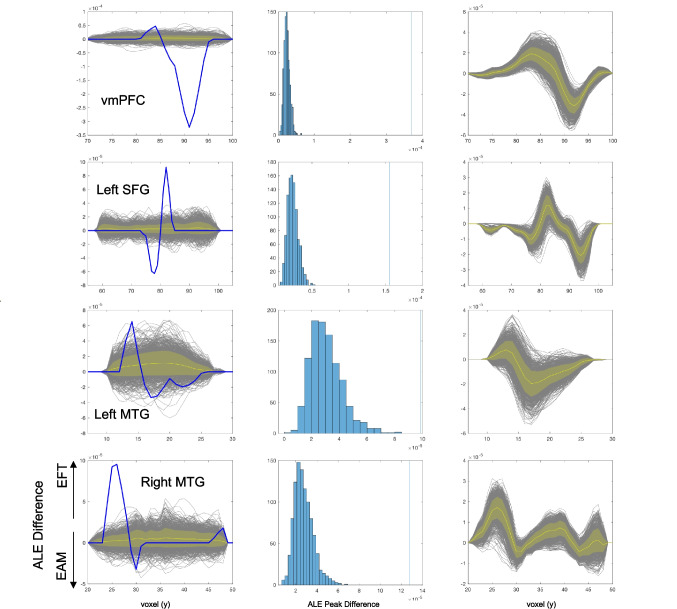
ALE gradient analysis. Left panels represent the ALE gradients observed for each of the four regions, displayed as blue traces. Gray lines represent the null distribution from 1000 random permutations of ALE coordinates for each map; yellow line and shaded region represent the mean and standard deviation. Accordingly, the yellow trace should not be similar to the blue trace for the gradient to not be due to random chance. Middle panels represent the peak-to-peak difference for the null distribution points in the left panel; the blue vertical line represents the peak-to-peak difference for the observed gradient, which would overlap with the null distribution if due to chance. Right panels display the gradients observed using 70% of ALE values for each map across 1000 permutations; yellow line and shaded regions again represent the mean and standard deviation. Accordingly, the permutation gradients seen in the right panels should be similar to the blue trace lines in the left panels if the gradients are stable

For the gradient analysis, we identified four regions where gradients were reliably observed: the left and right aMTG, left SFG, and vmPFC (Fig. 3 and Fig. 4). All other regions where intersections were found did not present reliable gradients. Among the four remaining regions, the left SFG and vmPFC presented the strongest evidence for a gradient, as both regions exhibited a gradient difference that well-exceeded the null distribution and also presented a gradient in the stability plots. However, the left SFG exhibited a stability plot with an additional negative deflection, suggesting the gradient here may have been due to a small number of studies. The right MTG similarly presented a stability plot that lacked a clear gradient, further suggesting this region does not reliably represent a true gradient across studies. This leaves the vmPFC as the most reliable region for a gradient, with anterior voxels representing EAM and posterior voxels representing EFT.

## Discussion

In this study, we performed a quantitative meta-analysis of fMRI studies assessing brain correlates of episodic autobiographical memory (EAM) and episodic future thinking (EFT), using Activation Likelihood Estimation (ALE). Our first aim was to provide an updated picture of neural networks commonly involved EAM and EFT, as well as to identify brain regions showing stronger activation for one of these two processes compared with the other. To these goals, as a preliminary step we performed two individual ALE meta-analyses to identify brain networks respectively supporting EAM and EFT. Secondarily, contrast analyses were performed between the EAM and EFT ALE maps, allowing to highlight brain regions more consistently activated by one of these two processes compared to the other. Convergence between the two ALE maps was also assessed, analyzing the degree of overlap between brain networks supporting EAM and EFT. Finally, we tested the possibility of a gradient transition between these two domains in regions of joint activation, to assess whether the two domains may be functionally organized in a topographical manner at the neural level along contiguous cortical regions.

### Brain Networks Supporting EAM and EFT

Consistently with previous findings (Boccia et al., 2019; Shepardson et al., 2023; Svoboda et al., 2006; Teghil et al., 2021), the meta-analysis on EAM highlighted a network involving the PCC/RSC and the left vmPFC on the medial brain surface, as well as the bilateral aMTG and AG, and the left IFG and SFG on the lateral surface. The PHG was also involved bilaterally, with activation extending to the HC in the right hemisphere. Further clusters of activation were highlighted in the right FG extending to the PHG and in the pCer. Concerning the individual meta-analysis on EFT, clusters of activation were found in the PCC and in the vmPFC as well as in the bilateral AG, aMTG and PHG, extending to the HC in the left hemisphere. The left SFG and the bilateral anterior cerebellum were also activated.

Notably, an ALE meta-analysis on EFT has been recently performed by Cona and colleagues (2023a) in a study assessing brain correlates of different types of future-oriented cognitive processes (future thinking, delay discounting and prospective memory). Our findings are generally consistent with those reported in that study, in which EFT was associated with activation of midline structures, including the PCC/pCu and the medial frontal cortex, as well as of the left AG and MTG. However, whereas the network highlighted by Cona and co-authors (2023a) was entirely left-lateralized, here we also observed clusters of activation in the right hemisphere, including the AG, aMTG and PHG, as well as further clusters in the left SFG and PHG/HC. Moreover, no hippocampal activation was detected for EFT in the Cona et al. (2023a) study. Whereas most contrasts included in Cona and colleagues (2023a) involved the comparison of EFT with EAM, here we adopted a more inclusive approach, considering contrasts between EFT and different types of high-level control conditions (see Table S2). It is thus possible that the lack of the involvement of the PHG/HC in this previous meta-analysis may result from the prevalence of contrasts comparing EFT with EAM, thus ruling out the contribution of the PHG/HC. Consistently with this possibility, both the results of the conjunction analysis between EAM and EFT performed in this study (see below) and those of a previous meta-analysis specifically assessing convergence between brain networks supporting EAM and EFT (Benoit & Schacter, 2015) pointed to the PHG/HC as shared regions between the two domains. Present findings thus suggest that the medial temporal lobes may generally support EFT and EAM, rather than being more involved in one process compared to the other (see below for further discussion of this point).

### Common and Specific Activations Between EFT and EAM

Our first aim was to assess commonalities and differences between brain regions supporting EAM and EFT. The conjunction analysis highlighted a network playing a role in both domains, including midline structures (PCC/RS and mOFG), as well as the bilateral AG, aMTG and PHG, and the left SFG. These results are in line with those by Benoit and Schacter (2015), identifying a core network overlapping with the DMN in the largest quantitative meta-analysis on EAM and EFT to date. Some regions previously implicated in the core network supporting episodic simulation, such as the right dorsolateral prefrontal cortex and the left IFG/insula (Benoit & Schacter, 2015), were not activated in the conjunction analysis in the present study. Activation of the right dorsolateral prefrontal cortex and of the IFG during episodic processing has been mainly associated with controlled retrieval and memory search, as well as with the manipulation and monitoring of retrieved memories (Cabeza & St Jacques, 2007; Gilboa, 2004; Svoboda et al., 2006). Our results suggest that the involvement of these regions in EAM and EFT may be more dependent on task demands, rather than being specifically associated with the imagination and memory of the personal past and future.

Results of the contrast analyses highlighted brain regions more strongly involved in EAM and EFT compared with the other domain. The only previous ALE study comparing brain activation patterns supporting EFT with those involved in EAM was performed on a relatively small number of contrasts, and also included experiments requiring the generation of fictitious, implausible or counterfactual episodes (Benoit & Schacter, 2015). While generating fictitious events and possible future ones involves shared process components, such as the combination of episodic detail, the construction of a spatially coherent scene and the reliance on schematic knowledge (Addis, 2020), differences between the two may also be expected, as only episodic future thinking entails chronestesia and the contextualization in time of constructed events. Thus, this is the first meta-analysis specifically providing information on which nodes support the generation of possible future personal events compared with past ones. In the present study, when contrasting EAM with EFT, no significant cluster was highlighted. The contrast EFT > EAM, instead, showed a single cluster of activation corresponding to the aMTG. The aMTG is part of the dorsal-medial subsystem of the DMN (Andrews-Hanna et al., 2010), that has been implicated in the retrieval of semantic and conceptual knowledge, as well as in narrative processing, and has been proposed to be involved in the emergence of verbal and inferential features of imagination (Andrews-Hanna et al., 2014; Andrews-Hanna & Grilli, 2021). The anterior part of the right middle temporal gyrus, specifically, has been shown to be activated during the imagination of future plausible compared with less plausible events (Roberts et al., 2017), and to be involved in supporting a conceptual orientation during autobiographical memory retrieval (Gurguryan & Sheldon, 2019). A key role of anterior middle temporal areas in EFT has been suggested by evidence that gray matter volume in these regions correlates with the generation of episodic future details (Irish et al., 2012a, 2013). Evidence of a stronger activation of the right aMTG during EFT compared with EAM is thus consistent with the semantic scaffolding hypothesis, suggesting that this region may be implicated in the retrieval of conceptual knowledge during the construction of future events (Irish et al., 2012a, 2012b; Irish & Piguet, 2013). The right aMTG has been shown to be involved in both the retrieval of personal episodes and the imagination of fictitious episodes about someone else (Rabin et al., 2010), showing stronger activation during the imagination of episodes involving personally known individuals compared with the recollection of one’s own life events (Rabin & Rosenbaum, 2012). Moreover, this region has been found to be involved in temporal sequencing of personal semantic information (Teghil et al., 2022). Thus, an interesting possibility to be tested in future studies is that the right aMTG may contribute to the simulation of personal future events supporting the retrieval of autobiographical knowledge and personal semantic information.

Within the most recent formulation of the constructive episodic simulation hypothesis, simulations of personal past and future events have been proposed to vary on a continuum of associative strength and schema reliance, corresponding to a differential involvement of regions of the DMN, of the anterior part of the DMN (aDMN) and of frontoparietal control networks (FPCN); based on this framework, the imagination of plausible and novel future events should entail very strong activation of DMN regions and moderately high activation of the aDMN/FPCN, whereas the recall of autobiographical events should correspond to moderate activation of the DMN and low involvement of the aDMN/FPCN, due to the lower requirement for schema reliance and conceptual processing (Addis, 2020). Present results are only partially consistent with this account, providing no evidence of stronger activation of regions such as the PHG/HC, the dorsomedial and dorsolateral PFC, the PCC and the lateral parietal cortex in EFT compared with EAM.

### Gradient Organization of EAM and EFT

Besides highlighting brain regions commonly and differentially supporting EAM and EFT, the present study aimed to test the possibility of a gradient transition between these two domains at the neural level. To this goal, the presence of functional gradients was tested in regions of conjunction between EFT and EAM. We found that the left SFG, the vmPFC and the bilateral aMTG all showed spatial gradients. Moreover, different patterns of gradients were observed in such regions. In more detail, the left SFG displayed a symmetrical, rostrocaudal gradient, with EAM and EFT activating respectively more posterior and more anterior regions. The vmPFC displayed a reverse pattern, with a rostrocaudal organization of the gradient favoring EAM in more anterior and EFT in more posterior regions. The gradient observed in this area appeared to favor EAM over EFT, as shown by its asymmetrical shape. Finally, gradients observed in the bilateral aMTG were organized along a rostrocaudal axis.

#### Shared Involvement of Parieto-medial Temporal Regions in EFT and EAM

Evidence for the presence of functional gradients in different regions commonly involved in EFT and EAM is consistent with the possibility that phenomenological and cognitive features of EAM and EFT may at least partly be accounted for by a gradual transition in the functional properties of involved brain regions. Notably, different regions highlighted by the conjunction analysis, including the PHG/HC and the AG, did not show any gradiental transition. According to the constructive episodic simulation hypothesis, a stronger involvement of the HC should be expected during EFT compared with EAM, reflecting the need for the retrieval of details from different past episodes, as well as the increased recombination demands supporting the generation of personal future events (Benoit & Schacter, 2015; Schacter & Addis, 2007). The anterior HC, specifically, has been further suggested to be more involved in the generation of future events, due to increased associative processes during episodic simulation (Addis, 2020; Addis & Schacter, 2012). Our results, showing a similar involvement of medial temporal lobe structures across EFT and EAM, appear not to be entirely consistent with this account. Importantly, differences in hippocampal activation between EAM and EFT have been reported in relation to the memory phase (event retrieval vs. construction) (Addis et al., 2007; Weiler et al., 2010). Since here we did not classify studies according to the memory phase, present results do not allow to exclude the possibility that this factor may consistently modulate the involvement of medial temporal lobes in EFT and EAM. Hippocampal involvement has been further found to be modulated by the specificity and novelty of the constructed event (Addis et al., 2011; van Mulukom et al., 2013). Nonetheless, all these factors cannot be considered specific to EAM or EFT per se, but rather reflect superordinate features of the simulated events, regardless from their temporal reference. Overall, present results concerning hippocampal involvement in EAM and EFT are in line with the proposal that both processes involve retrieval and recombination demands crucially subserved by the medial temporal lobes (Addis & Schacter, 2012; Schacter & Addis, 2007). However, they further suggest that specific features of EFT and EAM may be not primarily linked to a differential involvement of medial temporal lobes, and could possibly be explained by a differential involvement of component processes other than episodic detail retrieval/recombination. Similarly with the HC, no gradient was observed in the PCC/RSC or AG in the present meta-analysis. These regions are both part of a parieto-medial temporal pathway that has been implicated in learning and retrieval of navigational knowledge (Boccia et al., 2015, 2016). Moreover, the AG and the PCC are commonly activated by episodic autobiographical memory and environmental navigation tasks performed using an egocentric perspective (Teghil et al., 2021). These findings provide thus support to the scene construction theory, positing that the overlap between brain networks supporting EAM and EFT reflects the dependence of both processes on the construction and maintenance of a complex visuo-spatial event scene (Hassabis & Maguire, 2007; Maguire & Mullally, 2013). The PCC has been proposed to be involved in the retrieval of contextual features during both the recall of remembered and the simulation of imagined events (Szpunar et al., 2009). Supporting this possibility, imagined events can be decoded in this region based on their spatial context (Robin et al., 2018), and posterior cingulate regions appear to support orientation in different cognitive domains, including space and time (Peer et al., 2015). On the other hand, lesions to the AG impair free recall of autobiographical memories, leading to reduced re-experiencing of retrieved events (Berryhill et al., 2007). This is in line with evidence that multimodal episodic memories can be decoded in the AG, and that classification accuracy shows an association with the vividness of recollected events (Bonnici et al., 2016). The AG has been further implicated in autobiographical memory by evidence that continuous theta-burst stimulation of this region reduced the specificity of recalled events, as well as the tendency to re-experience such events from a first-person perspective (Bonnici et al., 2018), in line with the proposal that the AG has a key role in the phenomenological experience of relieving (Bréchet et al., 2018; Moscovitch et al., 2016). This account is further supported by evidence that inhibitory TMS to the left AG (in a region in close proximity to the left AG cluster highlighted by the conjunction analysis in the present study) reduces specificity for both recalled autobiographical and simulated personal future events and increases task difficulty (Thakral et al., 2017). Overall, evidence for a common involvement of the PCC and AG during EFT and EAM supports proposals that these regions may contribute to the two domains anchoring constructed simulations and memories to a spatiotemporal context (Ranganath & Ritchey, 2012), as well as supporting re- and pre-experiencing from a first-person perspective through the integration of event details within an egocentric frame of reference (Teghil et al., 2021, 2022; Bonnici et al., 2018; Bréchet et al., 2018; D’Argembeau, 2020).

#### Functional Gradients Supporting Transition Between EAM and EFT

Functional gradients characterizing the transition in common activations between EFT and EAM were highlighted in different regions, specifically in the left SFG, the vmPFC, and the bilateral aMTG.

A symmetrical gradient was observed in the left SFG, with more anterior regions preferentially supporting EFT and more posterior ones preferentially supporting EAM; the central portion of the left SFG, instead, was commonly activated. Gradient transitions have been previously described in the frontal lobes (Badre & D’Esposito, 2009; Abdallah et al., 2022). In more detail, a rostrocaudal functional gradient in the lateral prefrontal cortex has been shown to be associated with increasing level of abstraction (Badre & D’Esposito, 2009; Abdallah et al., 2022). Different subregions of the SFG have been shown to display a graded functional pattern, with further subregions identifiable along the antero-posterior axis, and the dorsal part showing an overall connectivity profile consistent with the possibility that this region may act as connection node between the DMN and the central-executive network (Abdallah et al., 2022; Li et al., 2013). Notably, whereas the middle portion of the lateral frontal cortex has been associated with overall contextual control of behavior, the more rostral and caudal parts have been respectively implicated in schema-related and sensorimotor processing (Badre & Nee, 2018). Within this framework, the gradient observed in the left SFG in the present study could reflect a domain-general role of this region in strategic and episodic retrieval (Svoboda et al., 2006; Stawarczyk & D’Argembeau, 2015). More rostral and caudal regions, instead, would be preferentially active for EFT and EAM, being the former more oriented towards schematic control, and the latter towards sensory processing (Badre & Nee, 2018), in line with the proposal that EFT is more dependent on the activation of schema knowledge compared with EAM (Addis, 2020; D’Argembeau, 2020; Ciaramelli et al., 2021) and with evidence that EAM entails a stronger reactivation of perceptual features compared with EFT (D’Argembeau and Van der Linden, 2004; 2006; Addis et al., 2009b).

A gradient of activation was further observed in the vmPFC. This gradient also showed a rostrocaudal progression, with more anterior and posterior regions respectively more involved in EAM and EFT. The medial prefrontal cortex has been widely implicated in self-referencing during both EFT and EAM (Svoboda et al., 2006; Martinelli et al., 2013; Stawarczyk & D’Argembeau, 2015; Kim, 2012). Based on the known role of the vmPFC in supporting instantiation and accommodation in relation to existing schemas (Ghosh & Gilboa, 2014; Gilboa & Marlatte, 2017), the medial prefrontal cortex has been proposed to contribute to episodic simulation through the activation of a self-related schema, allowing the integration of retrieved elements into a common personal framework, that features a representation of the content and structure of one’s life (Verfaellie et al., 2019; D’Argembeau, 2020). Notably, patients with lesions to the vmPFC are impaired not only in the retrieval and imagination of personal past/future events, but show similar deficits when asked to imagine events concerning other people (Bertossi et al., 2016a) or fictitious events not explicitly self-relevant (Bertossi et al., 2016b). These findings suggest that the role of the vmPFC in EAM and EFT may be not related to self-refencing per se, but rather to the ability to decouple from the external environment to generate simulations that combine episodic details into representations of novel events (Benoit et al., 2014; Bertossi et al., 2016a). The vmPFC shows graded anatomical and structural connectivity patterns, with the more dorsal/anterior part showing greater connectivity with regions of the DMN, including the PCC, AG and HC, and the more ventral/posterior extreme being more strongly connected with the ventral visual stream and the ventral part of the anterior temporal lobe (Jackson et al., 2020). Importantly, although both the retrieval of past and the simulation of future events entail a combination of semantic and episodic components (Addis et al., 2007; Greenberg & Rubin, 2003; Hassabis & Maguire, 2007), EFT relies more strongly on previous knowledge (Duval et al., 2012; Irish et al., 2012a, 2012b). The gradient observed in the present meta-analysis, with more ventral/posterior regions of the vmPFC favoring the processing of EFT, is thus in line with the abovementioned functional and anatomical findings. On this view, our results suggest that the role of the vmPFC in EAM and EFT may well be to support the reactivation of schemas, as well as the integration of retrieved elements into a coherent framework (Bertossi et al., 2016a; McCormick et al., 2018). Nonetheless, it is possible to hypothesize this role may be not completely overlapping in the EAM and EFT domains, with a greater predominance respectively of the recombination of episodic details (supported by more dorsal/anterior portions) and the integration of semantic components (supported by more ventral/posterior portions). Although this possibility needs to be supported by further studies, it would be in line with evidence of a fine-grained functional specialization of the mPFC (Benoit et al., 2012; Eickhoff et al., 2016a, 2016b; Gilbert et al., 2010) and with the proposal that this region may contribute differently to EAM and EFT (Benoit & Schacter, 2015; Bertossi et al., 2016a).

The bilateral aMTG also showed evidence of a gradient transition between activations for EAM and EFT. In both hemispheres, gradients were organized along a rostocaudal direction, with more anterior activation likelihood for EAM, although at a much lower degree than for EFT at posterior voxels. The organization of gradients was partially different between hemispheres, with EFT being represented more strongly in the right hemisphere. The lateral temporal cortex shows graded patterns of change in functional connectivity (Jackson et al., 2018), as well as different structural connectivity gradients, including a lateral-to-medial gradient, an anterolateral-to-posteromedial gradient and an antero-posterior gradient (Blazquez Freches et al., 2020; Vos de Wael et al., 2021). Different aMTG subregions have been reported to show distinguishable connectivity profiles, with a more ventral-anterior portion preferentially connected with the DMN and the FPCN, and a more dorsal-posterior part more connected with the sensorimotor and executive control network (Xu et al., 2019). The anterior part of the middle temporal gyrus has been mainly implicated in semantic processing (Battistella et al., 2019; Binder et al., 2009), although a relative hemispheric specialization has been proposed, with the left lateral temporal cortex preferentially involved in language and semantics, and the right in theory of mind and social knowledge processing (Blazquez Freches et al., 2020; Pobric et al., 2016). Lateral temporal cortex gradients have been proposed to reflect different streams of convergence in these regions, gradually allowing the integration of sensory-specific information into higher-level and more abstract representations (Binney et al., 2012; Blazquez Freches et al., 2020). These regions could thus participate to EAM and EFT providing high-order, conceptual information about life events (Sheldon et al., 2019). While the activation of conceptual knowledge is essential to support both the retrieval of past events and the simulation of future ones, evidence of a functional specialization of the lateral temporal cortex, together with present data on a gradiental organization of EAM and EFT in such regions, further suggests that the aMTG may contribute in a not completely superimposable manner to the two processes. It is important to point out, however, that gradients highlighted in the aMTG were less clearly organized compared to those observed in frontal conjunction regions (see below). Thus, further evidence is needed to support this possibility.

## Conclusions

Taken together, results of the present meta-analysis suggest that differences between EAM and EFT may be accounted for by both specific and common activation patterns. Moreover, such a contribution appears to arise at least partially through the organization of specific regions of common activation between EAM and EFT along topographical functional gradients.

The HC, PCC and AG appear to generally support the remembering of our past experiences and the imagination of future personal events, possibly through the retrieval and recombination of event details (Schacter & Addis, 2007), the integration of such details in first-person imaginal framework (Bonnici et al., 2018) and the anchoring of such representations on a personal timeline (Teghil et al., 2022). Other regions, however, may provide a more specific contribution to the two domains. Present findings are in line with proposals that a predominant scaffolding by conceptual-semantic knowledge is involved when simulating hypothetic future events (Irish & Piguet, 2013; Irish et al., 2012a, 2012b) and that regions of the lateral temporal cortex may be involved in the retrieval of abstract conceptual knowledge, and in approaching our episodic details from our memories from a more narrative, thematic perspective (Andrews-Hanna & Grilli, 2021). Furthermore, evidence of stronger activation of the right aMTG in EFT compared with EAM is consistent with recent findings showing that right lateral temporal regions may specifically be involved in supporting autobiographical knowledge (Teghil & Boccia, 2024; Teghil et al., 2022). This possibility would fit well within theoretical frameworks suggesting that the subjective experience of being projected in our personal future (in contrast with that characterizing the imagination of an atemporal scene or a fictitious or non-personal event) derives from the integration of the constructed event with goals and expectations on our lives (D’Argembeau, 2020). From this perspective, EAM and EFT both involve constructive simulation processes, supported by a “core” network involving the bilateral PHG/HC, the PCC/RSC and vmPFC, the left SFG, and the bilateral AG and aMTG. Nonetheless, results of the present meta-analysis are also consistent with proposals that specific features distinguishing EFT from EAM do not primarily reflect the increased need for recombination of details and control processes in the former (Benoit & Schacter, 2015), but possibly derive from the increased need for (personal) semantic scaffolding that is required when simulating personal events, that have not been already experienced in the past. Further studies will be needed to test this possibility, as well as to specify the role of personal vs. general semantic knowledge in supporting EFT.

The present meta-analysis has different limitations. First, our results are not informative on more subtle differences in brain activation patterns associated with EFT and EAM that may be associated with different stages of memory retrieval, e.g. construction and elaboration (Addis et al., 2007; Daviddi et al., 2023; Sheldon et al., 2019; Weiler et al., 2010). The choice to include contrasts reflecting both phases allowed us to highlight common and separate networks supporting EFT and EAM, independently from the specific paradigm or task phase (Müller et al., 2018). Also, the relatively low number of studies performed to date on EFT does not allow to test differences between retrieval stages. Nonetheless, understanding whether systematic differences in brain activity during the constructive process may be associated with EFT and EAM would be an important goal for future studies. It has been also highlighted that a key variable to consider when assessing behavioral and neural features of EFT and EAM concerns the temporal distance of retrieved/imagined events, as more distant memories are associated with different brain networks compared with recent ones (Boccia et al., 2019; Gilmore et al., 2021) and EFT and EAM are both affected by temporal distance (although with both symmetrical and asymmetrical effects) (D’Argembeau & Van der Linden, 2004; Colás‐Blanco et al., 2022). Whereas brain networks supporting remote and recent EAMs have been previously investigated using Activation Likelihood Estimation (Boccia et al., 2019), the low number of studies systematically considering the temporal distance of imagined events for the EFT domain did not allow to perform such an analysis.

Finally, in line with previous studies (Cona et al., 2021, 2023b), here we tested reliability and stability for each of the observed gradients. In the reliability analysis, the observed ALE difference exceeded the confidence interval for all gradients. However, a first caveat to be mentioned is that gradients in the bilateral aMTG appeared to be less reliable than those in the left SFG and vmPFC. Moreover, the bootstrapped distribution for the SFG gradient showed two negative peaks, suggesting that the direction of the gradient was at least partially affected by studies included in the meta-analysis. The gradient observed in the vmPFC, on the other hand, was not symmetrical, showing that ALE values were favored for EAM; this suggests that this feature of the gradient was not entirely stable. Nonetheless, a clear rostrocaudal gradient could be observed in this case, supporting the interpretation of the direction of the effect. Overall, present findings should be hopefully replicated in the future, possibly including more experiments in the EFT domain.

To conclude, our results provide evidence that differences between EAM and EFT may be understood in light of both common- and process-specific activations, and that further specificities may be accounted for by an organization of EAM and EFT along functional gradients in neighboring cortical regions, in line with previous evidence of axes of structural and functional variance as a key organizing principle in the human cortex (Cona et al., 2021, 2023b; Huntenburg et al., 2018). An important question to be addressed in future research will be whether such gradients in task-related activation also correspond to graded anatomical and functional connectivity patterns, and whether such patterns may account for specific features of EFT and EAM. Moreover, if phenomenological and neural signatures of EAM and EFT can be explained based on the graded contribution of different process components to a common domain-general simulation system (Addis, 2020), the development of controlled paradigms allowing to independently manipulate the requirement for these process components will be essential to shed light on how our brain support the experience of mentally traveling our past and future.

## Supplementary Information

Below is the link to the electronic supplementary material.

Supplementary file1 (PDF 349 KB)

## Data Availability

Results can be accessed at the following link: https://osf.io/epfhk/?view_only=7f08d01a1b5b4d75a359c645f2b7accc.
