# Supplementary material for: Cortical Gradients Support Mental Time Travel into the Past and Future: Evidence from Activation Likelihood Estimation Meta-analysis
Source: Neuropsychol Rev. 2025 May 1;36(1):25–42. doi: 10.1007/s11065-025-09662-w (PMC13279674; doi:10.1007/s11065-025-09662-w)

Cortical gradients support mental time travel into the past and future: evidence from Activation Likelihood Estimation meta-analysis

Neuropsychology Review

Alice Teghil[a,b*], Martin Wiener[c], Maddalena Boccia[a,b]

[a] Department of Psychology, "Sapienza" University of Rome, Rome, Italy
[b] Cognitive and Motor Rehabilitation and Neuroimaging Unit, IRCCS Fondazione Santa Lucia, Rome, Italy
[c] Department of Psychology, George Mason University, Fairfax, VA

*Corresponding author at:

Alice Teghil
Department of Psychology
"Sapienza" University of Rome
Via dei Marsi, 78, 00185 Rome, Italy
E-mail address: alice.teghil@uniroma1.it

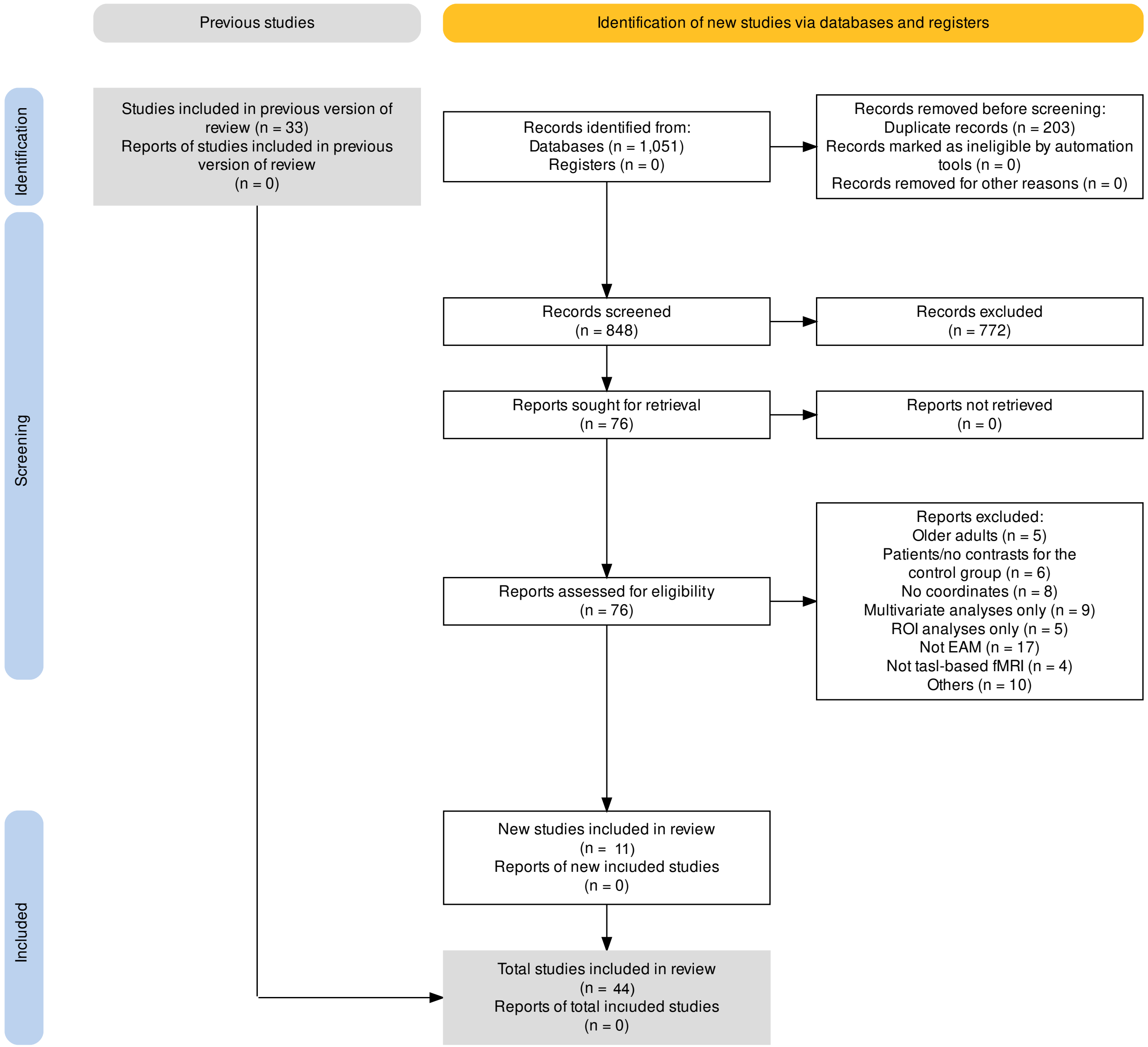

Figure S1. PRISMA 2020 flow diagram for episodic autobiographical memory (EAM). The flow diagram was generated using the Shiny app for producing PRISMA 2020-compliant flow diagrams (Haddaway et al., 2022).

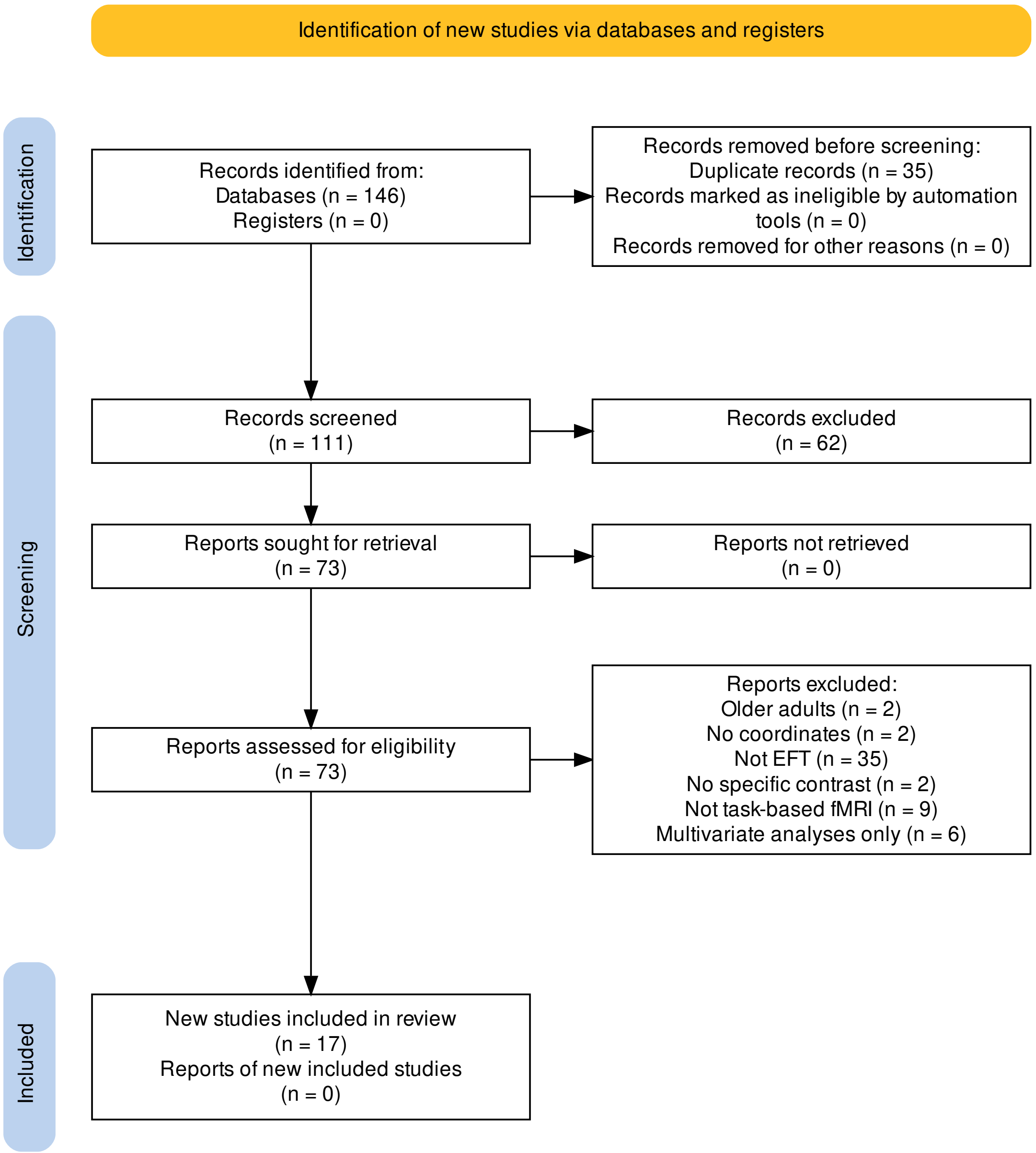

Figure S2. PRISMA 2020 flow diagram for episodic future thinking (EFT). The flow diagram was generated using the Shiny app for producing PRISMA 2020-compliant flow diagrams (Haddaway et al., 2022).

References

Haddaway, N. R., Page, M. J., Pritchard, C. C., & McGuinness, L. A. (2022). PRISMA2020: An R package and Shiny app for producing PRISMA 2020-compliant flow diagrams, with interactivity for optimised digital transparency and Open Synthesis Campbell Systematic Reviews, 18, e1230. https://doi.org/10.1002/cl2.1230

Article	Task	N° of contrasts	Contrast
Addis et al., 2004	Retrieval of previously collected EAMs from titles	1	EAM > sentence completion/size discrimination
Arshamian et al., 2013	Retrieval of previously collected EAMs from odors and odor-words cues	3	OEAM > control odors
			1st decade OEAM > 2nd decade OEAM
			EAM > control words
Audrain et al., 2022	Overt spoken recall of EAMs from scene photographs	2	Construction phase > Control task (picture description)
			Construction phase, temporal distance effect
Botzung et al., 2008a	Retrieval of previously collected EAMs from personalized word cues	1	EAM > SM
Botzung et al., 2008b	Retrieval of previously collected EAMs from word cues	3	EAM > SM
			Recent EAMs > remote EAMs
			Remote EAMs > recent EAMs
Cabeza et al., 2004	Recognition of photographs taken by the participant	1	Controlled EAM > controlled laboratory memory
Chen et al., 2017	Retrieval of EAMs from scene cues	3	Life memory, studied (hits) > unstudied (hits)
			Life memory ("yes" responses to novel scenes) > picture memory ("yes" responses to studie scenes)
			Life memory > picture memory ("yes" responses to studied scenes)
Compère et al., 2016	Retrieval of TPI-collected EAMs from personalized word cues	2	EAM > control imagery (men)
			EAM > control imagery (women)
Compère et al., 2018	Recall of EAMs/semantic autobiographical memories (SAM) from word cues	2	EAM > Definition (whole sample)
			EAM > SAM (whole sample)
Denkova et al., 2006	Retrieval of EAMs from photographs of friends and relatives	1	EAM > face identification
Detour et al., 2011	Retrieval of EAMs from personal photographs	2	[EAMs discrimination (hits) > discrimination (CRs)] U [observation (hits) > observation (CRs
			[Discrimination (hits − CRs) > Observation (hits − CRs)]
Donix et al., 2010	Retrieval of previously collected EAMs from personalized sentence cues	1	(EAM recent + EAM remote) > (SM recent + SM remote), young participants
Eich et al., 2009	Retrieval of EAMs of specific activities from icon cues	3	Increased BOLD response for field memories

			Increased BOLD response for field vs. observer memories
			Increased BOLD response for observer memories
Fleischer et al., 2019	Recall of EAMs from word cues	1	Recall > Calculate (placebo condition)
Ford et al., 2016	Retrieval of EAMs from song cues	1	Regions exhibiting a significant relation between neural activity and ratings of song familiarit young participants
Fuentes-Claramonte et al., 2019	Recall of EAMs from word cues vs. semantic completion	1	Memory > Control
Gardini et al., 2006	Generation of mental images of EAMs from word cues	2	EAM imagery > baseline (silent reading/button pressing)
			EAM imagery > exemplar imagery
Gilboa et al., 2004	Retrieval of EAMs from personal photographs	1	Vividly > non-vividly recollected events
Gilmore et al., 2021a	Overt spoken recall of EAMs from scene photographs	1	Temporal distance effect
Gilmore et al., 2021b	Overt spoken recall of EAMs from scene photographs	1	Autobiographical recall > picture description, sustained activity
Gilmore et al., 2018	Imagination/retrieval of FEs/EAMs and non-personal events from word cues	2	Remember > Other
			Remember > Future
Grol et al., 2017	Imagery of previosuly collected EAMs from personalized word cues	1	Imagery of memories > visual search
Gurguryan & Sheldon, 2019	Recall of EAMs from word focusing on conceptual/contextual details	2	Contextual > conceptual, re-oriented recollection stage
			Contextual > conceptual, oriented recollection stage
Hoscheidt et al., 2010	True/false decision on statements concerning previously collected EAMs	3	EAM > SM
			Non-spatial EAM > sentence reading
			Spatial EAM > sentence reading
Ino et al., 2011	Retrieval of EAMs from general event cues	1	(EAM > Rest) U (EAM> SM)
Lux et al., 2015	Retrieval of previously collected EAMs from personalized sentence cues	5	Location context > temporal context
			Temporal and location context (recent weighted stronger)
			Location contex (recent weighted stronger)
			Temporal context (recent weighted stronger)
			Temporal context > location context
	True/false decision on statements concerning previously collected EAMs	4	EAM > Autobiographical facts (young group)

Maguire & Frith, 2003			EAM > syllable decision
			EAM > General knowledge (young group)
			EAM > Public event memory (young group)
Masaoka et al., 2021	Recall of EAMs from odors	1	AM odor > Control odor
Monsa et al., 2020	Order judgment of EAMs	1	Temporal task > Lexical judgment control task
Muscatell et al., 2010	Retrieval of EAMs from word cues	1	EAM > control imagery
Niki & Luo, 2002	Retrieval of EAMs of visiting places from personalized landmark cues	2	Recent EAMs > remote EAMs
			Remote memories > recent memories
Noreen et al., 2016	Retrieval of specific aspects of previously collected EAMs from cue words	1	EAM > EAM suppression
Oddo et al., 2010	Retrieval of previously collected EAMs from personalized sentence cues	3	EAM > SM
			(Recent EAMs – remote EAMs) > (recent SMs– remote SMs)
			Recent EAMs > remote EAMs
Rabin et al., 2010	Retrieval of EAMs from personal photographs	3	EAM > ToM
			EAM > ToM construction
			EAM > ToM elaboration
Rekkas & Constable, 2005	Retrieval of EAMs from question cues	2	Recent EAMs > General knowledge
			Remote EAMs > General knowledge
St. Jacques et al., 2011	Retrieval of EAMs from verbal cues/photograph cues collected by SenseCam	1	(EAM SenseCam Cue - EAM Verbal Cue) males > (EAM SenseCam Cue - EAM Verbal Cu[illegible] females
St. Jacques et al., 2013	Retrieval of EAMs of specific events from photographs/Old-New recognition task	5	Target phase > lure phase
			True memories (target > lure) > false memories (target > lure)
			True memories > false memories
			Memory reliving, hits (target > lure) > false alarms (target > lure)
			Memory reliving, target phase > lure phase
St. Jacques et al., 2017	Retrieval of previously collected EAMs from titles (self and non-self perspective)	1	Repetition suppression effects
St. Jacques et al., 2018	Retrieval of previously collected EAMs from titles	2	EAM (initial Recall – identical Recall repetition)

			EAM > episodic counterfactual simulation
Summerfield et al., 2009	Retrieval of previously collected EAMs from personalized sentence cues	1	(EAM - Object memory) > (Imagined memory - Imagined object)
Svoboda & Levine, 2009	Retrieval of previously collected EAMs from personal recording cues	1	EAM reharsal (first reharsal)
Teghil et al., 2022	Temporal ordering of EAMs and experience-near personal semantic memories (enPS)	1	EAM > enPS
Terhoeven et al., 2023	Recall of EAMs from word cues	4	Specific food/body-related AM > food/body-related example generation (healthy controls)
			Specific neutral AM vs. neutral example generation (healthy controls)
			Specific food/body-related AM > unspecific food/body-related AM (healthy controls)
			Specific neutral AM > unspecific neutral AM (healthy controls)
Young et al., 2013	Retrieval of EAMs from word cues	1	EAM > Example generation (males + females)

Table S1. Articles included in the meta-analysis on episodic autobiographical memory (EAM). For each paper, details about the task, number of contrasts from each article and the specific contrasts included in the meta-analysis are reported. The reference list for the articles is provided below.
EAM = episodic autobiographical memories; FEs = future events; Lag = temporal window after stimulus onset (see original articles for further details); CR = correct rejection; SM = semantic memory; OEAM = olfactory episodic autobiographic memory; TIQ = Taste and Interest Questionnaire; ToM = Theory of mind; TR = temporal window after stimulus onset (see original articles for further details).

Addis, D. R., Moscovitch, M., Crawley, A. P., & McAndrews, M. P. (2004). Recollective qualities modulate hippocampal activation during autobiographical memory retrieval. Hippocampus, 14(6), 752–762. https://doi.org/10.1002/hipo.10215

Arshamian, A., Iannilli, E., Gerber, J.C., Willander, J., Persson, J., Seo, H.S., Hummel, T.,
Botzung, A., Denkova, E., & Manning, L. (2008b). Experiencing past and future personal events: functional neuroimaging evidence on the neural bases of mental time travel. Brain and cognition, 66(2), 202–212. https://doi.org/10.1016/j.bandc.2007.07.011

Audrain, S., Gilmore, A. W., Wilson, J. M., Schacter, D. L., & Martin, A. (2022). A Role for the Anterior Hippocampus in Autobiographical Memory Construction Regardless of Temporal Distance. The Journal of neuroscience : the official journal of the Society for Neuroscience, 42(33), 6445–6452. https://doi.org/10.1523/JNEUROSCI.0832-22.2022

Botzung, A., Denkova, E., Ciuciu, P., Scheiber, C., & Manning, L. (2008a). The neural bases of the constructive nature of autobiographical memories studied with a self-paced fMRI design. Memory (Hove, England), 16(4), 351–363. https://doi.org/10.1080/09658210801931222

Botzung, A., Denkova, E., & Manning, L. (2008b). Experiencing past and future personal events: functional neuroimaging evidence on the neural bases of mental time travel. Brain and cognition, 66(2), 202–212. https://doi.org/10.1016/j.bandc.2007.07.011

Cabeza, R., Prince, S. E., Daselaar, S. M., Greenberg, D. L., Budde, M., Dolcos, F., LaBar, K. S., & Rubin, D. C. (2004). Brain activity during episodic retrieval of autobiographical and laboratory events: an fMRI study using a novel photo paradigm. Journal of cognitive neuroscience, 16(9), 1583–1594. https://doi.org/10.1162/0898929042568578

Chen, H. Y., Gilmore, A. W., Nelson, S. M., & McDermott, K. B. (2017). Are There Multiple Kinds of Episodic Memory? An fMRI Investigation Comparing Autobiographical and Recognition Memory Tasks. The Journal of neuroscience : the official journal of the Society for Neuroscience, 37(10), 2764–2775. https://doi.org/10.1523/JNEUROSCI.1534-16.2017

Compère, L., Rari, E., Gallarda, T., Assens, A., Nys, M., Coussinoux, S., Machefaux, S., & Piolino, P. (2018). Gender identity better than sex explains individual differences in episodic and semantic components of autobiographical memory and future thinking. Consciousness and cognition, 57, 1–19. https://doi.org/10.1016/j.concog.2017.11.001

Compère, L., Sperduti, M., Gallarda, T., Anssens, A., Lion, S., Delhommeau, M., Martinelli, P., Devauchelle, A. D., Oppenheim, C., & Piolino, P. (2016). Sex Differences in the Neural Correlates of Specific and General Autobiographical Memory. Frontiers in human neuroscience, 10, 285. https://doi.org/10.3389/fnhum.2016.00285

Denkova, E., Botzung, A., Scheiber, C., & Manning, L. (2006). Implicit emotion during recollection of past events: a nonverbal fMRI study. Brain research, 1078(1), 143–150. https://doi.org/10.1016/j.brainres.2006.01.061

Detour, J., Danion, J. M., Gounot, D., Marrer, C., & Foucher, J. R. (2011). Prefrontal cortex recruitment during naturalistic remote memory: a factorial block-event fMRI study. Brain research, 1400, 66–77. https://doi.org/10.1016/j.brainres.2011.05.024

Donix, M., Poettrich, K., Weiss, P. H., Werner, A., von Kummer, R., Fink, G. R., & Holthoff, V. A. (2010). Age-dependent differences in the neural mechanisms supporting long-term declarative memories. Archives of clinical neuropsychology : the official journal of the National Academy of Neuropsychologists, 25(5), 383–395. https://doi.org/10.1093/arclin/acq037

Eich, E., Nelson, A. L., Leghari, M. A., & Handy, T. C. (2009). Neural systems mediating field and observer memories. Neuropsychologia, 47(11), 2239–2251. https://doi.org/10.1016/j.neuropsychologia.2009.02.019

Fleischer, J., Metz, S., Düsenberg, M., Grimm, S., Golde, S., Roepke, S., Renneberg, B., Wolf, O. T., Otte, C., & Wingenfeld, K. (2019). Neural correlates of glucocorticoids effects on autobiographical memory retrieval in healthy women. Behavioural brain research, 359, 895–902. https://doi.org/10.1016/j.bbr.2018.06.024

Ford, J. H., Rubin, D. C., & Giovanello, K. S. (2016). The effects of song familiarity and age on phenomenological characteristics and neural recruitment during autobiographical memory retrieval. Psychomusicology, 26(3), 199–210. https://doi.org/10.1037/pmu0000152

Fuentes-Claramonte, P., Martín-Subero, M., Salgado-Pineda, P., Alonso-Lana, S., Moreno-Alcázar, A., Argila-Plaza, I., Santo-Angles, A., Albajes-Eizagirre, A., Anguera-Camós, M., Capdevila, A., Sarró, S., McKenna, P. J., Pomarol-Clotet, E., & Salvador, R. (2019). Shared and differential default-mode related patterns of activity in an autobiographical, a self-referential and an attentional task. PloS one, 14(1), e0209376. https://doi.org/10.1371/journal.pone.0209376

Gardini, S., Cornoldi, C., De Beni, R., & Venneri, A. (2006). Left mediotemporal structures mediate the retrieval of episodic autobiographical mental images. NeuroImage, 30(2), 645–655. https://doi.org/10.1016/j.neuroimage.2005.10.012

Gilboa, A., Winocur, G., Grady, C. L., Hevenor, S. J., & Moscovitch, M. (2004). Remembering our past: functional neuroanatomy of recollection of recent and very remote personal events. Cerebral cortex (New York, N.Y. : 1991), 14(11), 1214–1225. https://doi.org/10.1093/cercor/bhh082

Gilmore, A. W., Nelson, S. M., Chen, H. Y., & McDermott, K. B. (2018). Task-related and resting-state fMRI identify distinct networks that preferentially support remembering the past and imagining the future. Neuropsychologia, 110, 180–189. https://doi.org/10.1016/j.neuropsychologia.2017.06.016

Gilmore, A. W., Quach, A., Kalinowski, S. E., González-Araya, E. I., Gotts, S. J., Schacter, D. L., & Martin, A. (2021a). Evidence supporting a time-limited hippocampal role in retrieving autobiographical memories. Proceedings of the National Academy of Sciences of the United States of America, 118(12), e2023069118. https://doi.org/10.1073/pnas.2023069118

Gilmore, A. W., Quach, A., Kalinowski, S. E., Gotts, S. J., Schacter, D. L., & Martin, A. (2021b). Dynamic Content Reactivation Supports Naturalistic Autobiographical Recall in Humans. The Journal of neuroscience : the official journal of the Society for Neuroscience, 41(1), 153–166. https://doi.org/10.1523/JNEUROSCI.1490-20.2020

Grol, M., Vingerhoets, G., & De Raedt, R. (2017). Mental imagery of positive and neutral memories: A fMRI study comparing field perspective imagery to observer perspective imagery. Brain and cognition, 111, 13–24. https://doi.org/10.1016/j.bandc.2016.09.014

Gurguryan, L., & Sheldon, S. (2019). Retrieval orientation alters neural activity during autobiographical memory recollection. NeuroImage, 199, 534–544. https://doi.org/10.1016/j.neuroimage.2019.05.077

Hoscheidt, S. M., Nadel, L., Payne, J., & Ryan, L. (2010). Hippocampal activation during retrieval of spatial context from episodic and semantic memory. Behavioural brain research, 212(2), 121–132. https://doi.org/10.1016/j.bbr.2010.04.010

Ino, T., Nakai, R., Azuma, T., Kimura, T., & Fukuyama, H. (2011). Brain activation during autobiographical memory retrieval with special reference to default mode network. The open neuroimaging journal, 5, 14–23. https://doi.org/10.2174/1874440001105010014

Lux, S., Bindrich, V. N., Markowitsch, H. J., & Fink, G. R. (2015). Medial temporal lobe activation during autobiographical context memory retrieval of time and place and its dependency upon recency. Neurocase, 21(1), 23–32. https://doi.org/10.1080/13554794.2013.860174

Maguire, E. A., & Frith, C. D. (2003). Aging affects the engagement of the hippocampus during autobiographical memory retrieval. Brain : a journal of neurology, 126(Pt 7), 1511–1523. https://doi.org/10.1093/brain/awg157

Masaoka, Y., Sugiyama, H., Yoshida, M., Yoshikawa, A., Honma, M., Koiwa, N., Kamijo, S., Watanabe, K., Kubota, S., Iizuka, N., Ida, M., Ono, K., & Izumizaki, M. (2021). Odors Associated With Autobiographical Memory Induce Visual Imagination of Emotional Scenes as Well as Orbitofrontal-Fusiform Activation. Frontiers in neuroscience, 15, 709050. https://doi.org/10.3389/fnins.2021.709050

Monsa, R., Peer, M., & Arzy, S. (2020). Processing of Different Temporal Scales in the Human Brain. Journal of cognitive neuroscience, 32(11), 2087–2102. https://doi.org/10.1162/jocn_a_01615

Muscatell, K. A., Addis, D. R., & Kensinger, E. A. (2010). Self-involvement modulates the effective connectivity of the autobiographical memory network. Social cognitive and affective neuroscience, 5(1), 68–76. https://doi.org/10.1093/scan/nsp043

Niki, K., & Luo, J. (2002). An fMRI study on the time-limited role of the medial temporal lobe in long-term topographical autobiographic memory. Journal of cognitive neuroscience, 14(3), 500–507. https://doi.org/10.1162/089892902317362010

Noreen, S., O'Connor, A. R., & MacLeod, M. D. (2016). Neural Correlates of Direct and Indirect Suppression of Autobiographical Memories. Frontiers in psychology, 7, 379. https://doi.org/10.3389/fpsyg.2016.00379

Oddo, S., Lux, S., Weiss, P. H., Schwab, A., Welzer, H., Markowitsch, H. J., & Fink, G. R. (2010). Specific role of medial prefrontal cortex in retrieving recent autobiographical memories: an fMRI study of young female subjects. Cortex; a journal devoted to the study of the nervous system and behavior, 46(1), 29–39. https://doi.org/10.1016/j.cortex.2008.07.003

Rabin, J. S., Gilboa, A., Stuss, D. T., Mar, R. A., & Rosenbaum, R. S. (2010). Common and unique neural correlates of autobiographical memory and theory of mind. Journal of cognitive neuroscience, 22(6), 1095–1111. https://doi.org/10.1162/jocn.2009.21344

Rekkas, P. V., & Constable, R. T. (2005). Evidence that autobiographic memory retrieval does not become independent of the hippocampus: an fMRI study contrasting very recent with remote events. Journal of cognitive neuroscience, 17(12), 1950–1961. https://doi.org/10.1162/089892905775008652

St Jacques, P. L., Carpenter, A. C., Szpunar, K. K., & Schacter, D. L. (2018). Remembering and imagining alternative versions of the personal past. Neuropsychologia, 110, 170–179. https://doi.org/10.1016/j.neuropsychologia.2017.06.015

St Jacques, P. L., Conway, M. A., & Cabeza, R. (2011). Gender differences in autobiographical memory for everyday events: retrieval elicited by SenseCam images versus verbal cues. Memory (Hove, England), 19(7), 723–732. https://doi.org/10.1080/09658211.2010.516266

St Jacques, P. L., Olm, C., & Schacter, D. L. (2013). Neural mechanisms of reactivation-induced updating that enhance and distort memory. Proceedings of the National Academy of Sciences of the United States of America, 110(49), 19671–19678. https://doi.org/10.1073/pnas.1319630110

St Jacques, P. L., Szpunar, K. K., & Schacter, D. L. (2017). Shifting visual perspective during retrieval shapes autobiographical memories. NeuroImage, 148, 103–114. https://doi.org/10.1016/j.neuroimage.2016.12.028

Summerfield, J. J., Hassabis, D., & Maguire, E. A. (2009). Cortical midline involvement in autobiographical memory. NeuroImage, 44(3), 1188–1200. https://doi.org/10.1016/j.neuroimage.2008.09.033

Svoboda, E., & Levine, B. (2009). The effects of rehearsal on the functional neuroanatomy of episodic autobiographical and semantic remembering: a functional magnetic resonance imaging study. The Journal of neuroscience : the official journal of the Society for Neuroscience, 29(10), 3073–3082. https://doi.org/10.1523/JNEUROSCI.3452-08.2009

Teghil, A., Bonavita, A., Procida, F., Giove, F., & Boccia, M. (2022). Temporal Organization of Episodic and Experience-near Semantic Autobiographical Memories: Neural Correlates and Context-dependent Connectivity. Journal of cognitive neuroscience, 34(12), 2256–2274. https://doi.org/10.1162/jocn_a_01906

Terhoeven, V., Nikendei, C., Faschingbauer, S., Huber, J., Young, K. D., Bendszus, M., Herzog, W., Friederich, H. C., & Simon, J. J. (2023). Neurophysiological correlates of disorder-related autobiographical memory in anorexia nervosa. Psychological medicine, 53(3), 844–854. https://doi.org/10.1017/S003329172100221X

Young, K. D., Bellgowan, P. S., Bodurka, J., & Drevets, W. C. (2013). Functional neuroimaging of sex differences in autobiographical memory recall. Human brain mapping, 34(12), 3320–3332. https://doi.org/10.1002/hbm.22144

Article	Task	N° of contrasts	Contrast
Addis et al., 2011	Imagination/retrieval of FEs/EAMs from word cues	1	Future > past autobiographical tasks
Addis & Schacter, 2008	Imagination/retrieval of FEs/EAMs from word cues	1	Differential past–future responses to detail
Beaty et al., 2018	Imagination of FEs/sentence construction from word cues	1	Future > Sentence
Botzung et al., 2008	Retrieval of previously collected FEs from word cues	1	Increases in brain activity associated with future events evocation
Bulganin & Wittmann, 2015	Imagination of FEs for rewarded or non-rewarded (Neut) words vs. semantic control (Con) condition	2	Neut > Con, Construction phase
			Neut > Con, Elaboration phase
D'Argembeau et al., 2010	Imagination of previously collected FEs (personally relevant vs. personally not-relevant)/Routine activities from sentence cues	2	Personal Future Events > Routine Activities
			Nonpersonal Future Events > Routine Activities
Gaesser et al., 2013	Imagination of FEs for the first time/already imagined the day before from word cues vs. Size judgment	1	Re-imagine + Imagine > Size Control
Gilmore et al., 2018	Imagination/retrieval of FEs/EAMs and non-personal events from word cues	2	Future > Remember
			Future > Other
Madore et al., 2016	Imagination of FE following specific/non-specific detail cueing vs. Generation of a sentence with two objects	4	Imagine construction > object construction for control induction
			Imagine construction > object construction for specificity induction
			Imagine elaboration > object elaboration for control induction
			Imagine elaboration > object elaboration for specificity induction
Martin et al., 2011	Imagination of FEs from word cues vs. Construction of a sentence about objects size	2	Regions activated by successful future event encoding
			Regions activated by imagining future events relative to the control condition
Roberts et al., 2017	Imagination of FEs from word cues with details from the same set (Congruent)/from different sets (Incongruent)	4	Incongruent > Congruent (TR 2)
			Congruent > Incongruent (TR 2)
			Incongruent > Congruent (TR 4)
			Congruent > Incongruent (TR 4)
Stillman et al., 2017	Imagination of FEs set tomorrow (Temporal proximity)/in 5 years (temporal distance)	2	Temporal distance > Temporal proximity
			Temporal proximity > Temporal distance
Thakral et al., 2017a	Imagination of FEs from word cues	2	Transient effects
			Sustained effects

Thakral et al., 2017b	Imagination of FEs from word cues	2	Transient simulation effects
			Sustained simulation effects
Van Hoeck et al., 2013	Imagination/retrieval of FEs/EAMs/counterfactual events from word cues	2	Future > Past
			Future > Counter
Weiler et al. 2010a	Imagination/retrieval of FEs/EAMs from word cues	2	Future > Past, Construction phase
			Future > Past, Elaboration phase
Weiler et al. 2010b	Imagination/retrieval of FEs/EAMs from word cues	1	Coordinates of Significantly Activated Clusters of the Future Thinking Condition Irrespective of Modulating Effects

Table S2. Articles included in the meta-analysis on episodic future thinking (EFT). For each paper, details about the task, number of contrasts from each article and the specific contrasts included in the meta-analysis are reported. The reference list for the articles is provided below.
FEs = future events; EAMs = episodic autobiographical memories.

Addis, D. R., Cheng, T., Roberts, R. P., & Schacter, D. L. (2011). Hippocampal contributions to the episodic simulation of specific and general future events. Hippocampus, 21(10), 1045–1052. https://doi.org/10.1002/hipo.20870

Addis, D. R., & Schacter, D. L. (2008). Constructive episodic simulation: temporal distance and detail of past and future events modulate hippocampal engagement. *Hippocampus*, *18*(2), 227–237. https://doi.org/10.1002/hipo.20405

Beaty, R. E., Thakral, P. P., Madore, K. P., Benedek, M., & Schacter, D. L. (2018). Core Network Contributions to Remembering the Past, Imagining the Future, and Thinking Creatively. Journal of cognitive neuroscience, 30(12), 1939–1951. https://doi.org/10.1162/jocn_a_01327

Bulganin, L., & Wittmann, B. C. (2015). Reward and Novelty Enhance Imagination of Future Events in a Motivational-Episodic Network. PloS one, 10(11), e0143477. https://doi.org/10.1371/journal.pone.0143477

Botzung, A., Denkova, E., & Manning, L. (2008). Experiencing past and future personal events: functional neuroimaging evidence on the neural bases of mental time travel. Brain and cognition, 66(2), 202–212. https://doi.org/10.1016/j.bandc.2007.07.011

D'Argembeau, A., Stawarczyk, D., Majerus, S., Collette, F., Van der Linden, M., Feyers, D., Maquet, P., & Salmon, E. (2010). The neural basis of personal goal processing when envisioning future events. *Journal of cognitive neuroscience*, *22*(8), 1701–1713. https://doi.org/10.1162/jocn.2009.21314

Gaesser, B., Spreng, R. N., McLelland, V. C., Addis, D. R., & Schacter, D. L. (2013). Imagining the future: evidence for a hippocampal contribution to constructive processing. Hippocampus, 23(12), 1150–1161. https://doi.org/10.1002/hipo.22152

Gilmore, A. W., Nelson, S. M., Chen, H. Y., & McDermott, K. B. (2018). Task-related and resting-state fMRI identify distinct networks that preferentially support remembering the past and imagining the future. Neuropsychologia, 110, 180–189. https://doi.org/10.1016/j.neuropsychologia.2017.06.016

Madore, K. P., Szpunar, K. K., Addis, D. R., & Schacter, D. L. (2016). Episodic specificity induction impacts activity in a core brain network during construction of imagined future experiences. *Proceedings of the National Academy of Sciences of the United States of America*, *113*(38), 10696–10701. https://doi.org/10.1073/pnas.1612278113

Martin, V. C., Schacter, D. L., Corballis, M. C., & Addis, D. R. (2011). A role for the hippocampus in encoding simulations of future events. Proceedings of the National Academy of Sciences of the United States of America, 108(33), 13858–13863. https://doi.org/10.1073/pnas.1105816108

Roberts, R. P., Wiebels, K., Sumner, R. L., van Mulukom, V., Grady, C. L., Schacter, D. L., & Addis, D. R. (2017). An fMRI investigation of the relationship between future imagination and cognitive flexibility. Neuropsychologia, 95, 156–172. https://doi.org/10.1016/j.neuropsychologia.2016.11.019

Stillman, P. E., Lee, H., Deng, X., Unnava, H. R., Cunningham, W. A., & Fujita, K. (2017). Neurological evidence for the role of construal level in future-directed thought. Social cognitive and affective neuroscience, 12(6), 937–947. https://doi.org/10.1093/scan/nsx022

Thakral, P. P., Benoit, R. G., & Schacter, D. L. (2017a). Characterizing the role of the hippocampus during episodic simulation and encoding. Hippocampus, 27(12), 1275–1284. https://doi.org/10.1002/hipo.22796

Thakral, P. P., Benoit, R. G., & Schacter, D. L. (2017b). Imagining the future: The core episodic simulation network dissociates as a function of timecourse and the amount of simulated information. Cortex; a journal devoted to the study of the nervous system and behavior, 90, 12–30. https://doi.org/10.1016/j.cortex.2017.02.005

Van Hoeck, N., Ma, N., Ampe, L., Baetens, K., Vandekerckhove, M., & Van Overwalle, F. (2013). Counterfactual thinking: an fMRI study on changing the past for a better future. Social cognitive and affective neuroscience, 8(5), 556–564. https://doi.org/10.1093/scan/nss031

Weiler, J. A., Suchan, B., & Daum, I. (2010a). When the future becomes the past: Differences in brain activation patterns for episodic memory and episodic future thinking. Behavioural brain research, 212(2), 196–203. https://doi.org/10.1016/j.bbr.2010.04.013

Weiler, J. A., Suchan, B., & Daum, I. (2010b). Foreseeing the future: occurrence probability of imagined future events modulates hippocampal activation. Hippocampus, 20(6), 685–690. https://doi.org/10.1002/hipo.206

Table S3 - PRISMA 2020 Main Checklist

Topic	No.	Item	Location where item is reported
**TITLE**			
**Title**	1	Identify the report as a systematic review.	1
**ABSTRACT**			
**Abstract**	2	See the PRISMA 2020 for Abstracts checklist	
**INTRODUCTION**			
**Rationale**	3	Describe the rationale for the review in the context of existing knowledge.	3-4
**Objectives**	4	Provide an explicit statement of the objective(s) or question(s) the review addresses.	4-5
**METHODS**			
**Eligibility criteria**	5	Specify the inclusion and exclusion criteria for the review and how studies were grouped for the syntheses.	5-6
**Information sources**	6	Specify all databases, registers, websites, organisations, reference lists and other sources searched or consulted to identify studies. Specify the date when each source was last searched or consulted.	5
**Search strategy**	7	Present the full search strategies for all databases, registers and websites, including any filters and limits used.	5
**Selection process**	8	Specify the methods used to decide whether a study met the inclusion criteria of the review, including how many reviewers screened each record and each report retrieved, whether they worked independently, and if applicable, details of automation tools used in the process.	5-6
**Data collection process**	9	Specify the methods used to collect data from reports, including how many reviewers collected data from each report, whether they worked independently, any processes for obtaining or confirming data from study investigators, and if applicable, details of automation tools used in the process.	5-6

Topic	No.	Item	Location where item is reported
**Data items**	10a	List and define all outcomes for which data were sought. Specify whether all results that were compatible with each outcome domain in each study were sought (e.g. for all measures, time points, analyses), and if not, the methods used to decide which results to collect.	5-6
	10b	List and define all other variables for which data were sought (e.g. participant and intervention characteristics, funding sources). Describe any assumptions made about any missing or unclear information.	NA
**Study risk of bias assessment**	11	Specify the methods used to assess risk of bias in the included studies, including details of the tool(s) used, how many reviewers assessed each study and whether they worked independently, and if applicable, details of automation tools used in the process.	5-6, File S4
**Effect measures**	12	Specify for each outcome the effect measure(s) (e.g. risk ratio, mean difference) used in the synthesis or presentation of results.	6-7
**Synthesis methods**	13a	Describe the processes used to decide which studies were eligible for each synthesis (e.g. tabulating the study intervention characteristics and comparing against the planned groups for each synthesis (item 5)).	5-6
	13b	Describe any methods required to prepare the data for presentation or synthesis, such as handling of missing summary statistics, or data conversions.	6
	13c	Describe any methods used to tabulate or visually display results of individual studies and syntheses.	7-12
	13d	Describe any methods used to synthesize results and provide a rationale for the choice(s). If meta-analysis was performed, describe the model(s), method(s) to identify the presence and extent of statistical heterogeneity, and software package(s) used.	6-7
	13e	Describe any methods used to explore possible causes of heterogeneity among study results (e.g. subgroup analysis, meta-regression).	NA
	13f	Describe any sensitivity analyses conducted to assess robustness of the synthesized results.	NA
**Reporting bias assessment**	14	Describe any methods used to assess risk of bias due to missing results in a synthesis (arising from reporting biases).	NA
**Certainty assessment**	15	Describe any methods used to assess certainty (or confidence) in the body of evidence for an outcome.	NA

Topic	No.	Item	Location where item is reported
**RESULTS**			
**Study selection**	16a	Describe the results of the search and selection process, from the number of records identified in the search to the number of studies included in the review, ideally using a flow diagram.	5-6; Figure S1; Figure S2
	16b	Cite studies that might appear to meet the inclusion criteria, but which were excluded, and explain why they were excluded.	NA
**Study characteristics**	17	Cite each included study and present its characteristics.	5-6; Table S1; Table S2
**Risk of bias in studies**	18	Present assessments of risk of bias for each included study.	File S4
**Results of individual studies**	19	For all outcomes, present, for each study: (a) summary statistics for each group (where appropriate) and (b) an effect estimate and its precision (e.g. confidence/credible interval), ideally using structured tables or plots.	NA
**Results of syntheses**	20a	For each synthesis, briefly summarise the characteristics and risk of bias among contributing studies.	NA
	20b	Present results of all statistical syntheses conducted. If meta-analysis was done, present for each the summary estimate and its precision (e.g. confidence/credible interval) and measures of statistical heterogeneity. If comparing groups, describe the direction of the effect.	7-12
	20c	Present results of all investigations of possible causes of heterogeneity among study results.	NA
	20d	Present results of all sensitivity analyses conducted to assess the robustness of the synthesized results.	NA
**Reporting biases**	21	Present assessments of risk of bias due to missing results (arising from reporting biases) for each synthesis assessed.	NA
**Certainty of evidence**	22	Present assessments of certainty (or confidence) in the body of evidence for each outcome assessed.	NA
**DISCUSSION**			
**Discussion**	23a	Provide a general interpretation of the results in the context of other evidence.	12-20
	23b	Discuss any limitations of the evidence included in the review.	20

Topic	No.	Item	Location where item is reported
	23c	Discuss any limitations of the review processes used.	20
	23d	Discuss implications of the results for practice, policy, and future research.	19-20
**OTHER INFORMATION**			
**Registration and protocol**	24a	Provide registration information for the review, including register name and registration number, or state that the review was not registered.	5-6
	24b	Indicate where the review protocol can be accessed, or state that a protocol was not prepared.	5-6
	24c	Describe and explain any amendments to information provided at registration or in the protocol.	NA
**Support**	25	Describe sources of financial or non-financial support for the review, and the role of the funders or sponsors in the review.	Title page
**Competing interests**	26	Declare any competing interests of review authors.	Title page
**Availability of data, code and other materials**	27	Report which of the following are publicly available and where they can be found: template data collection forms; data extracted from included studies; data used for all analyses; analytic code; any other materials used in the review.	Title page

PRIMSA Abstract Checklist

Topic	No.	Item	Reported?
**TITLE**			
**Title**	1	Identify the report as a systematic review.	Yes
**BACKGROUND**			
**Objectives**	2	Provide an explicit statement of the main objective(s) or question(s) the review addresses.	Yes
**METHODS**			
**Eligibility criteria**	3	Specify the inclusion and exclusion criteria for the review.	No
**Information sources**	4	Specify the information sources (e.g. databases, registers) used to identify studies and the date when each was last searched.	No
**Risk of bias**	5	Specify the methods used to assess risk of bias in the included studies.	No
**Synthesis of results**	6	Specify the methods used to present and synthesize results.	Yes
**RESULTS**			
**Included studies**	7	Give the total number of included studies and participants and summarise relevant characteristics of studies.	No
**Synthesis of results**	8	Present results for main outcomes, preferably indicating the number of included studies and participants for each. If meta-analysis was done, report the summary estimate and confidence/credible interval. If comparing groups, indicate the direction of the effect (i.e. which group is favoured).	Yes
**DISCUSSION**			
**Limitations of evidence**	9	Provide a brief summary of the limitations of the evidence included in the review (e.g. study risk of bias, inconsistency and imprecision).	No
**Interpretation**	10	Provide a general interpretation of the results and important implications.	Yes
**OTHER**			
**Funding**	11	Specify the primary source of funding for the review.	No
**Registration**	12	Provide the register name and registration number.	No

From: Page MJ, McKenzie JE, Bossuyt PM, Boutron I, Hoffmann TC, Mulrow CD, et al. The PRISMA 2020 statement: an updated guideline for reporting systematic reviews. MetaArXiv. 2020, September 14. DOI: 10.31222/osf.io/v7gm2. For more information, visit: www.prisma-statement.org

File S4 – Risk of bias assessment of included studies

Adapted checklist for risk of bias in individual studies included in Activation Likelihood Estimation (ALE)

<u>Domain 1: Study population</u>

Signaling questions:

1. Was the participant population clearly described, including key demographics (e.g., age, gender, handedness)? <u>*Yes/No/Unclear*</u>
2. Were participants appropriately selected to represent the population? <u>*Yes/No/Unclear*</u>
3. Was sample size large enough to ensure robust activation findings? <u>*Yes/No/Unclear*</u>

Could the selection of participants have introduced bias? RISK: LOW/HIGH/UNCLEAR

<u>Domain 2: Imaging acquisition and analysis</u>

Signaling Questions:

1. Was the imaging protocol clearly described (e.g., scanner type, acquisition parameters)? <u>*Yes/No/Unclear*</u>
2. Were preprocessing steps clearly described? <u>*Yes/No/Unclear*</u>
3. Were appropriate statistical methods used to identify activation (e.g., thresholds, corrections for multiple comparisons)? <u>*Yes/No/Unclear*</u>

Could acquisition and analyses procedures have introduced bias? RISK: LOW/HIGH/UNCLEAR

<u>Domain 3: Task design and relevance to the meta-analysis</u>

Signaling Questions:

1. Was the experimental task or paradigm thoroughly described and relevant to the ALE meta-analysis question? <u>*Yes/No/Unclear*</u>
2. Were tasks sufficiently comparable to those in other studies included in the ALE meta-analysis? <u>*Yes/No/Unclear*</u>
3. Did the task design minimize potential confounding factors (e.g., cognitive load differences, variability in stimuli)? <u>*Yes/No/Unclear*</u>

Could the inclusion of the contrast have introduced bias? RISK: LOW/HIGH/UNCLEAR

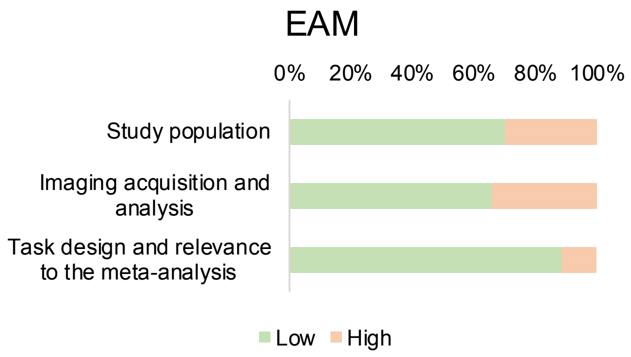

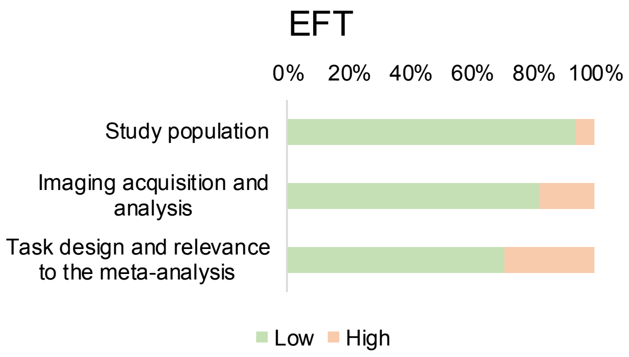

Supplement: Supplementary file 1 — Supplementary file1 (PDF 349 KB) [file 11065_2025_9662_MOESM1_ESM.pdf]
